# Repercussion of Solid state *vs*. Liquid state synthesized p-n heterojunction RGO-copper phosphate on proton reduction potential in water

**DOI:** 10.1038/s41598-018-21239-7

**Published:** 2018-02-13

**Authors:** Alaka Samal, Dipti P. Das, Giridhar Madras

**Affiliations:** 1 Academy of Scientific and Innovative Research, CSIR- Institute of Minerals and Materials Technology, Bhubaneswar, 751013 Odisha India; 2 Colloids and Material Chemistry, CSIR- Institute of Minerals and Materials Technology, Bhubaneswar, 751013 Odisha India; 3 Solid State and Structural Chemistry Unit, Indian Institute of Science, Bangalore, 560 012 India

## Abstract

The same copper phosphate catalysts were synthesized by obtaining the methods involving solid state as well as liquid state reactions in this work. And then the optimised p-n hybrid junction photocatalysts have been synthesized following the same solid/liquid reaction pathways. The synthesized copper phosphate photocatalyst has unique rod, flower, caramel-treat-like morphology. The Mott-Schottky behavior is in accordance with the expected behavior of n-type semiconductor and the carrier concentration was calculated using the M-S analysis for the photocatalyst. And for the p-n hybrid junction of 8RGO-Cu_3_(PO_4_)_2_-PA (PA abbreviated for photoassisted synthesis method), 8RGO-Cu_3_(PO_4_)_2_-EG(EG abbreviated for Ethylene Glycol based synthesis method), 8RGO-Cu_3_(PO_4_)_2_-PEG (PEG abbreviated for Poly(ethylene glycol)-*block*-poly(propylene glycol)-*block*-poly(ethylene glycol based synthesis method)the amount of H_2_ synthesized was 7500, 6500 and 4500 µmol/h/g, respectively. The excited electrons resulting after the irradiation of visible light on the CB of p-type reduced graphene oxide (RGO) migrate easily to n-type Cu_3_(PO_4_)_2_ *via*. the p-n junction interfaces and hence great charge carrier separation was achieved.

## Introduction

When coming to the reaction of natural photosynthesis, the role of terminal phosphate bond containing compound directly comes into the mind. These high energy terminal phosphates drive the chemical reactions in the synthesis of other biomolecules during the photosynthesis reaction steps. And this is why the photosynthesis process is curtailed without the phosphate as a catalyst. Alike the natural photosynthesis the artificial photosynthesis which converts the solar energy into chemical energy does need a best catalyst and should undergo a charge carrier transfer mechanism where it should face the least charge carrier recombination in presence of light. As the charge carriers unfortunately face recombination which ultimately obstacles the efficiency of catalysts due to shorter life time of the charge carriers.

To minimize the charge carriers recombination besides metal/semiconductor and carbon group materials/semiconductor-hetero- structured photocatalysts, semiconductor/semiconductor-heterostructured photocatalysts with diverse models have been developed including type-I and type-II heterojunctions, Z-scheme, p-n heterojunctions, and homojunction band alignments^1–4^. Apart from the design of other heterojunction photocatalytic systems, The construction of p-n junction between n-type and p-type narrow band gap semiconductors is an effective way to improve the photocatalytic reactivity, as the external electric field of p-n junction benefits the separation of electron-hole and enhances the photocatalytic capability dramatically^5–7^. RGO/GO is eminent p-type semiconductor photocatalysts because of oxygen’s high electronegativity compared to that of carbon atoms^8–13^. Many research was also dedicated on the advantages of the incorporation of graphene oxide/reduced graphene oxide (GO/RGO) sheets in the various semiconductors matrix but unfortunately, the role of graphene oxide/reduced graphene oxide (GO/RGO) is still very controversial and insufficient in the literature. Some work of RGO based composite suggest that the RGO can facilitate electron injection and assist in electron transport^14–16^, whereas other studies have reported that RGO can act as a sensitizer in semiconductor oxide films^17–19^. Recently, Na-intercalated graphene was used for production of hydrogen bubbles in water, resulting in self-propulsion of graphene particles in water^20^. Our group has successfully demonstrated the use of RGO as an individual semiconductor photocatalyst and designed in various heterojunction system *Ca*. Z-scheme, staggered heterojunction system etc, and this heterojunction systems showing great photocatalytic activity too^21–23^.

Well previously some researchers also designed the p-n heterojunction greatly enhances the charge generation and suppresses the charge recombination as well. Previously Wu *et al*., has designed p-n heterojunction by depositing p-type MoS_2_ nano-platelets on the n-type nitrogen-doped reduced graphene oxide (n-rGO) nanosheets for hydrogen generation^24^. Chung *et al*. also evaluated the semiconductor behavior of GO and RGO thin films upon formation of p-n junctions on n-type Si(111)^25^. Graphene oxide/TiO_2_ p/n Heterojunction composites in degradation of methyl orange was also studied bu Chen *et al*.^26^. And some other works focused on particular p-n junction formation with RGO are reported^27,28^. In this work, RGO is used as a p-type coupling semiconductor to n-type copper phosphate for the first time to achieve high visible light response. The photogenerated electron-hole pairs will be separated effectively by the p-n junction formed at the p-RGO/n-Cu_3_(PO_4_)_2_ interface. Also, we herein bequest a progression of inorganic copper phosphate micrometer-scale structures such as rod, brick, flowers, In chase of efficient solar-to-fuel conversion systems based on natural photosynthesis concept of using a phosphate catalyst.

## Result and Discussion

### Morphology and structural Analysis

**Figure 1:**
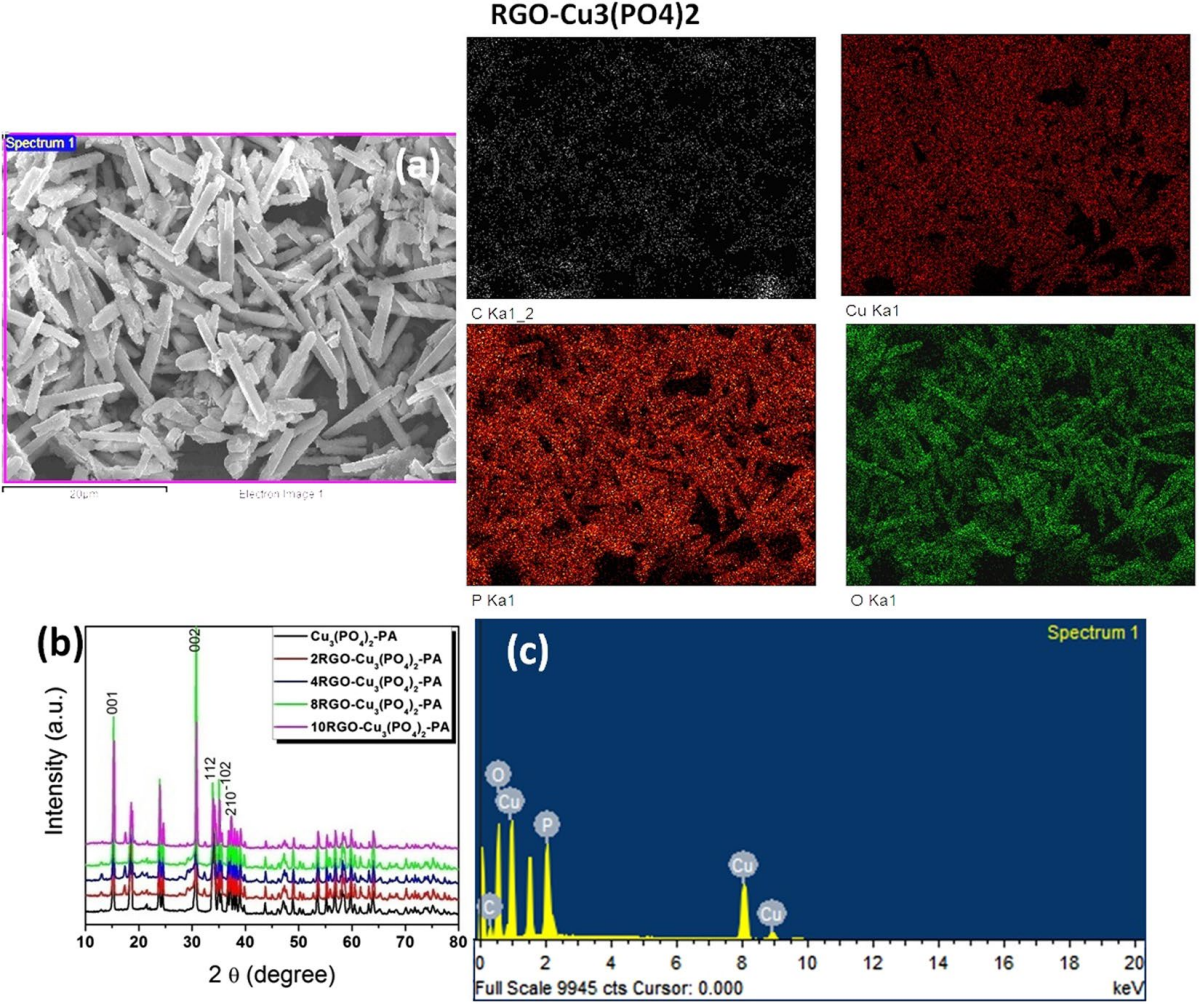
(**a**) The x-ray mapping of elements present in 8RGO-Cu_3_(PO_4_)_2_-PA. (**b**) XRD pattern of xRGO-Cu_3_(PO_4_)_2_-PA samples where x is 0,2,4,8,10. (**c**) EDX data of 8RGO-Cu_3_(PO_4_)_2_-PA collected by rastering the incident electron beam on the selected area with elemental composition.

The EDX technique was used to determine the composition of catalysts as a whole as well as the composition of individual components along with the x-ray mapping of elements present in 8RGO-Cu_3_(PO_4_)_2_-PA. Here the positions of specific elements emitting characteristic x-rays within an inspection field can be indicated by unique color. In Fig. 1a the maps of C, Cu, P and O are shown individually and overlaid with the original image. While in this elemental analysis specific elements are associated with specific areas. Elemental mapping is also showing the positions of inclusions of the present elements and also showing no contaminants in the said catalyst prepared by photo-assisted method. X-rays that have adequate energy to escape the material surface can be detected, resulting in a spectrum with peaks at the characteristic energies for the elements present. The areas under selected peaks can also be used to provide semi-quantitative elemental composition information. Considering in Fig. 1c shows an inspection field within which EDX data were collected by rastering the incident electron beam to produce the spectrum shown. The results reveal that C, Cu, P and O are the main elements present within the inspected field evidences the occurrence of the Cu_3_P_2_O_8_ composition in the selected area. Again the C element presence flaunts the existence of RGO in it. Figure 1b is the XRD pattern of the xRGO-Cu_3_(PO_4_)_2_-PA with the variables of x being 0, 2, 4, 8, 10 of weight percentages of RGO. The Expert high score software was used to validate the XRD patterns of the synthesized materials in the experiments. The patterns reveals the anorthic crystal system of copper phosphate with the compositional formula being Cu_3_(PO_4_)_2_ is well crystallized with the space group P-1. The lattice parameters with lattice constants values are obtained as a = 4.8537 Å, b = 5.2855 Å, and c = 6.1821 Å. And the crystal system angles are α = 72.3500°, β = 86.9900°, γ = 68.5400° (reference code: 01-070-0494).

**Figure 2:**
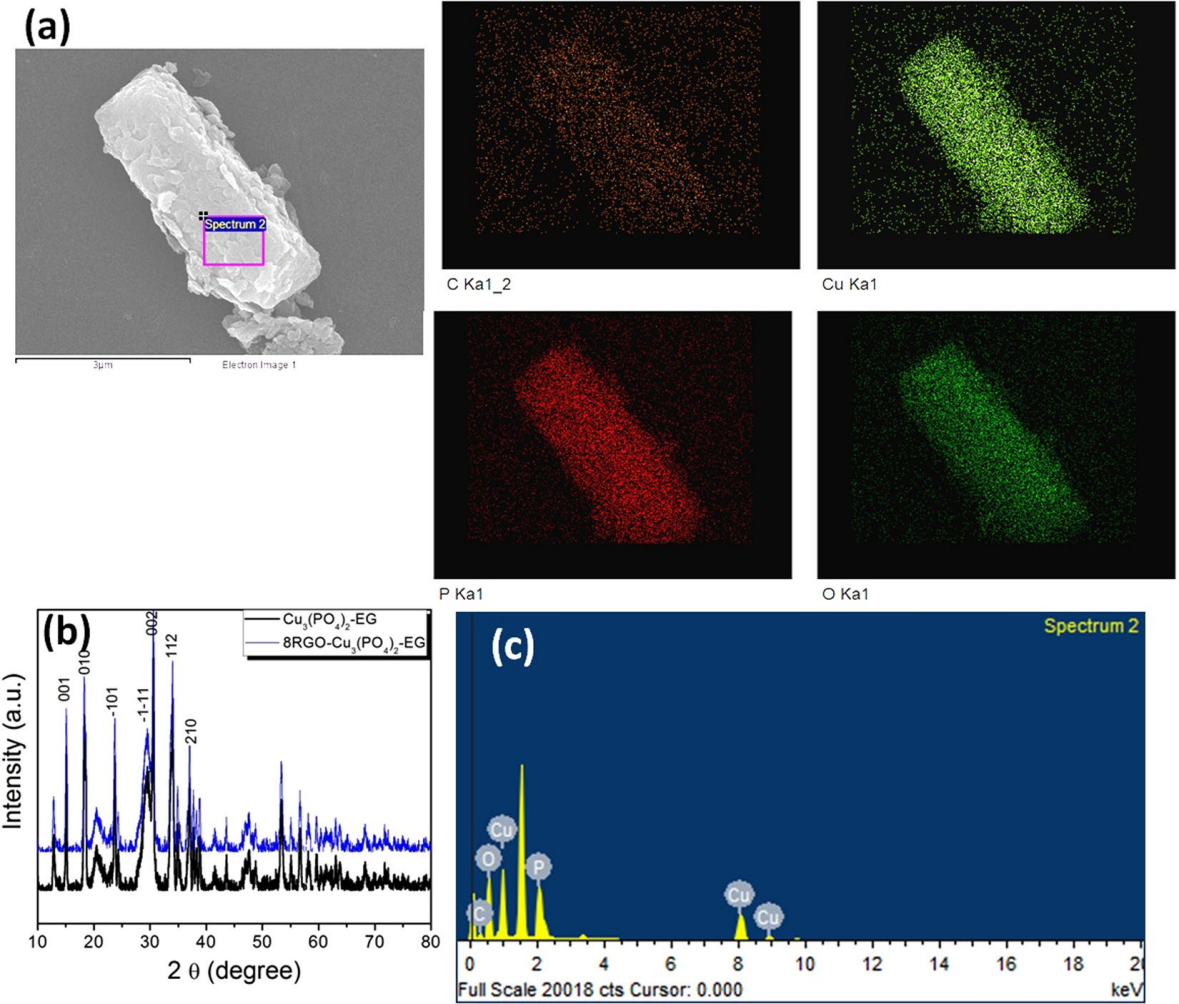
(**a**) The x-ray mapping of elements present in 8RGO-Cu_3_(PO_4_)_2_-EG. (**b**) XRD pattern of 8RGO-Cu_3_(PO_4_)_2_-EG and Cu_3_(PO_4_)_2_-EG samples. (**c**) EDX data of 8RGO-Cu_3_(PO_4_)_2_-EG collected by rastering the incident electron beam on the selected area with elemental composition.

Again the same elemental investigation evaluated for the ethylene glycol solvent mediated synthesized catalyst 8RGO-Cu_3_(PO_4_)_2_-EG. As shown in Fig. 2a, the elemental maps of C, Cu, P and O are shown individually and overlaid with the original image. While in this elemental analysis specific elements are associated with specific areas. Elemental mapping here also showing no contaminants in the said catalyst prepared by ethylene glycol as the solvent method. The EDX spectrum (Fig. 2c) shows that the catalyst as well exposing the main elements C, Cu, P and O as the focal elements as within the scrutinized field. The XRD pattern of corresponding catalyst also showing sharp diffraction peaks of Cu_3_(PO_4_)_2_ phase, testifying that it crystallizes well during the synthesis process in Fig. 2b. Alike the photoassisted synthesized catalyst this catalyst also showing the (001) and (002) planes at 2θ value 15.06° and 30.387°, respectively. This XRD pattern also reveals the anorthic crystal system of copper phosphate with the compositional formula being Cu_3_(PO_4_)_2_ is well crystallized with the space group P-1 along with the invariable lattice parameters values.

**Figure 3:**
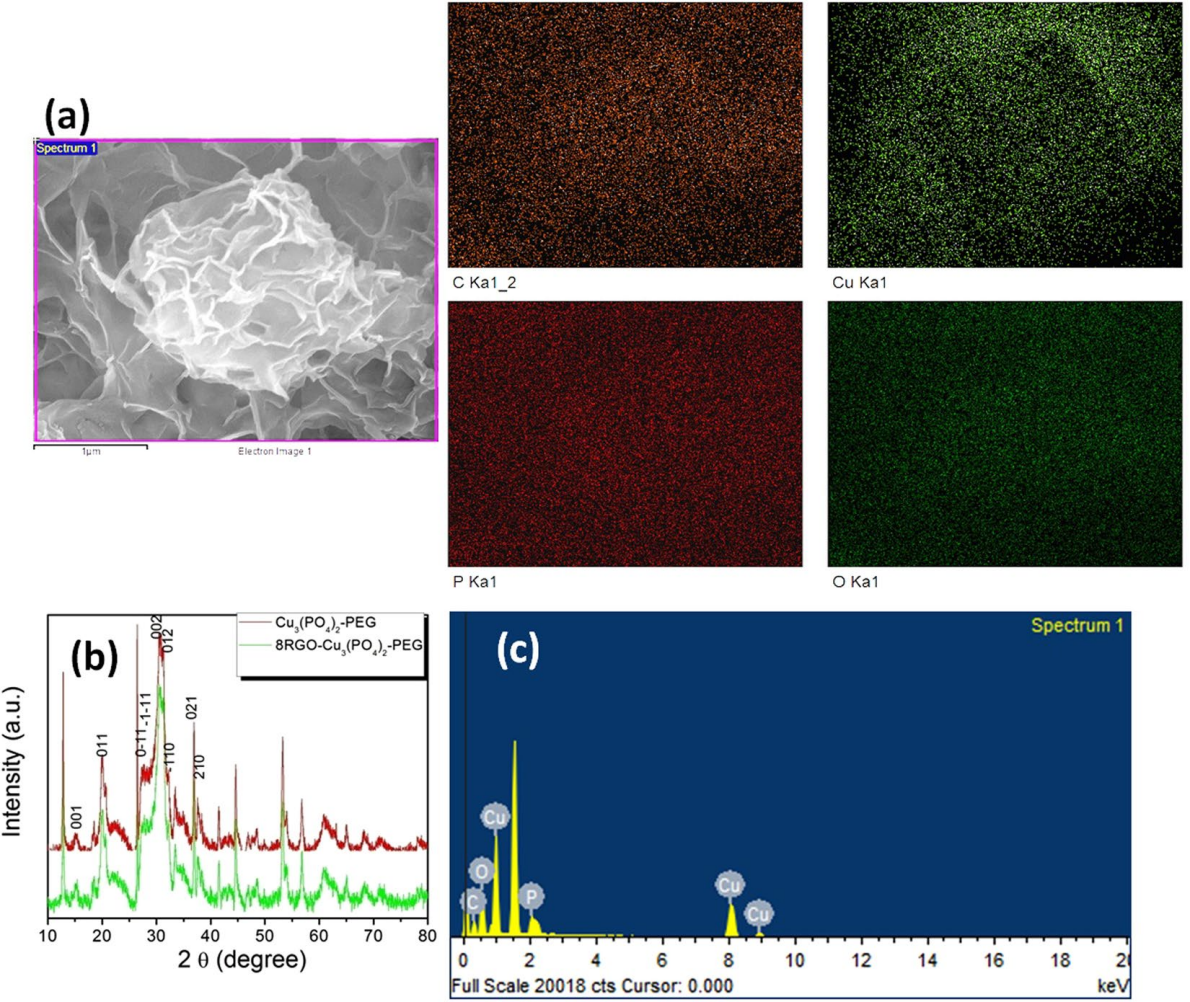
(**a**) The x-ray mapping of elements present in 8RGO-Cu_3_(PO_4_)_2_-PEG. (**b**) XRD pattern of 8RGO-Cu_3_(PO_4_)_2_-PEG and Cu_3_(PO_4_)_2_-PEG samples. (**c**) EDX data of 8RGO-Cu_3_(PO_4_)_2_-PEG collected by rastering the incident electron beam on the selected area with elemental composition.

The Elemental composition data from solid state synthesized catalyst also obtained and indexed in the Fig. 3a. This colored image is well defining the information about the distributions of Cu, P, O atoms and its associations in samples with RGO as C mapping confirming the well decorated successful synthesis of RGO-Cu_3_(PO_4_)_2_-PEG catalyst by a solid state reaction course. Solid state synthesized catalyst 8RGO-Cu_3_(PO_4_)_2_-PEG EDX data also collected by the same method. The prominent elements were C, Cu, P and O elements as within the scrutinized field evidence the occurrence of the Cu_3_P_2_O_8_ composition in the selected area in Fig. 3c. In Fig. 3b, series of well-defined (001), (011), (002), (012), (021) and (0–11) diffraction peaks are indexed to the anorthic phase (space group *P*-1) without any trace of other phase of copper phosphate. The XRD pattern of Cu_3_(PO_4_)_2_-PEG can be perfectly indexed to anorthic phase with the same lattice parameter values as the liquid state synthesized catalysts as above. After hybridizing with the RGO, the XRD pattern was found to be similar to that of Cu_3_(PO_4_)_2_-PEG due to the low content of RGO in it.

**Figure 4:**
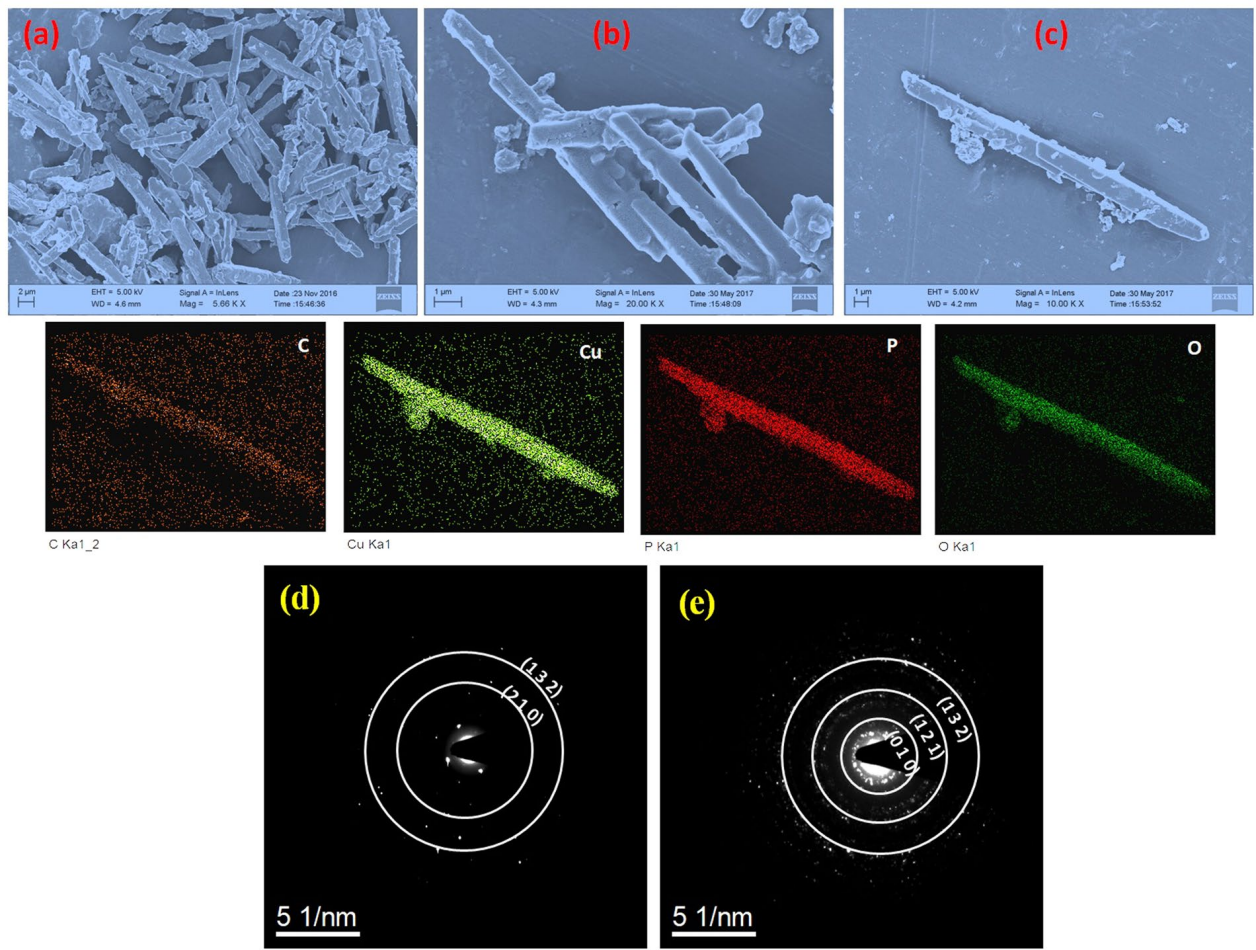
FE-SEM morphological analysis of (**a**) Cu_3_(PO_4_)_2_-PA, (**b**) 8RGO-Cu_3_(PO_4_)_2_-PA and (**c**) FE-SEM micrograph of single rod structure in 8RGO-Cu_3_(PO_4_)_2_-PA along with its elemental mapping. SAED patterns of (**d**) Cu_3_(PO_4_)_2_-PA (**e**) 8RGO-Cu_3_(PO_4_)_2_-PA.

In the morphological study of the catalysts fabricated by way of liquid state reaction under photo-irradiation, the morphology imaging surprisingly reveals an aggregation of rods with the average sizes of length of 7–10 µm and width of 0.6–0.7 µm (Fig. 4a,b). In the matter of pure and RGO hybrid catalyst the morphology is coming to be similar as expected. This rod of several micrometers in length was synthesized by taking a catalytic amount of the inexpensive triethanolamine. A single rod imaging was taken in the Fig. 4c showing a distinct form of a rod of the catalyst. The elemental mapping of the rod were taken by using the EDX characterization system shows uniform distribution of the C, Cu, P and O atoms evenly on the rod showing the exact formation of the hybrid with the RGO even in a single rod morphology. Furthermore, the crystallinity of the catalyst was confirmed by the SAED pattern as shown in Fig. 4d for Cu_3_(PO_4_)_2_-PA, and Fig. 4d for 8RGO-Cu_3_(PO_4_)_2_-PA. Analysis of ring patterns in crystalline materials leads to identification of phases in materials. Also, XRD analysis is used to determine the Miller indices for a set of planes. So, XRD analysis here confirms the results of diffraction pattern from TEM. The ring patterns is correspond to the (210), (132) planes of Cu_3_(PO_4_)_2_-PA. And in the case of 8RGO-Cu_3_(PO_4_)_2_-PA the ring patterns is corresponds to the (010), (121) and also for (132) planes. Astonishingly, in the SAED pattern of Cu_3_(PO_4_)_2_-PA exhibiting single crystalline structure but the catalyst with RGO *Ca*. 8RGO-Cu_3_(PO_4_)_2_-PA exhibiting polycrystalline structure.

**Figure 5:**
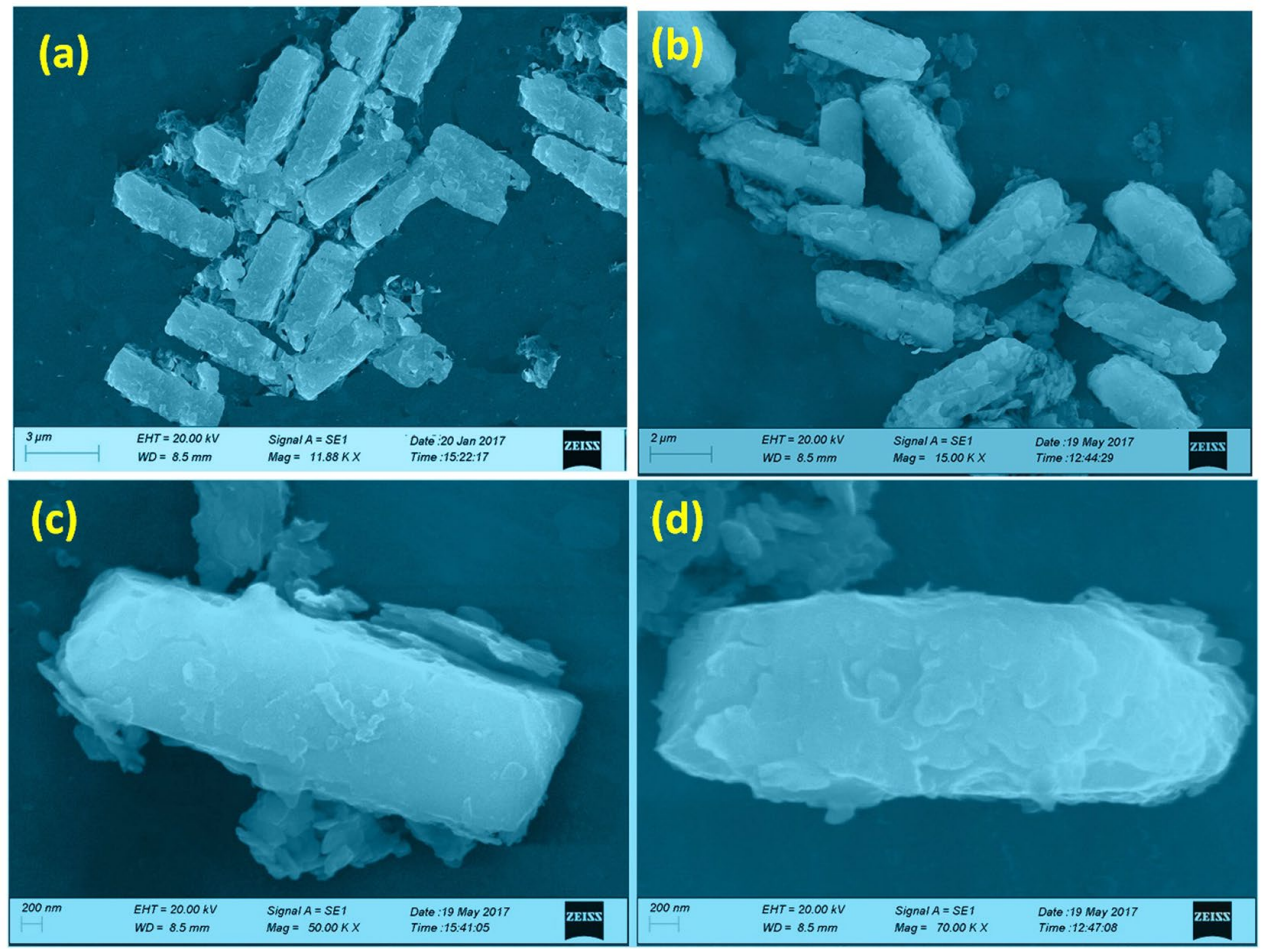
Low magnification FE-SEM images of (**a**) Cu_3_(PO_4_)_2_-EG and (**b**) 8RGO-Cu_3_(PO_4_)_2_-EG, high magnification SEM images of (**c**) Cu_3_(PO_4_)_2_-EG and (**d**) 8RGO-Cu_3_(PO_4_)_2_-EG showing “caramel-treat-like morphology.

The non aqueous based synthesis of the catalysts, Cu_3_(PO_4_)_2_-EG and 8RGO-Cu_3_(PO_4_)_2_-EG morphology data is given in Fig. 5a–d. It was observed that beautiful caramel-treat-like morphology is forming in the way of liquid state synthesis of the catalyst while ethylene glycol is the solvent for Cu_3_(PO_4_)_2_-EG. For 8RGO-Cu_3_(PO_4_)_2_-EG photocatalytic sample also the morphology was observed to be caramel-treat like with an extra flakes like structure on it. It can be evident that the caramel-treat-like structure maybe benefitted from the ethylene glycol medium and consequently this solvent environment giving rise a one directional morphological structure to the catalyst during the nucleation time. Figure 5a–d confirms that the unique one directional morphology with uniform particle size of length containing about 5.5 µm and width around 1.5 µm are prepared by means of this solvent synthesis method.

**Figure 6:**
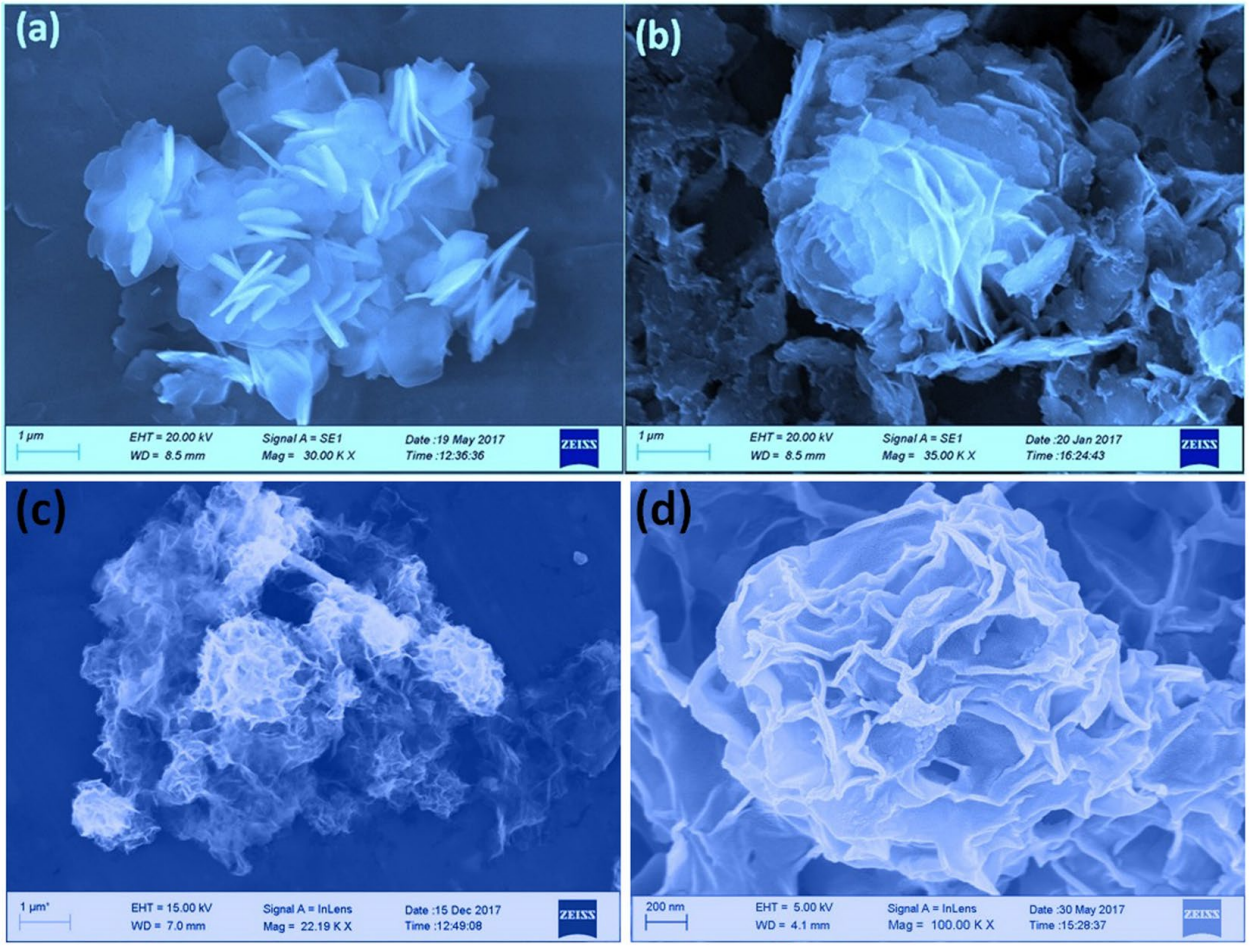
FE-SEM analysis of (**a**,**b**) Cu_3_(PO_4_)_2_-PEG synthesized by solid state reaction. (**c**,**d**) 8RGO-Cu_3_(PO_4_)_2_-PEG synthesized by solid state reaction with directly adding RGO during the grinding process.

Moreover the Cu_3_(PO_4_)_2_-PEG dispersions in isopropanol were dropped on aluminum foil, and arbitrarily many of FE-SEM images were taken for each sample. FE-SEM images of Cu_3_(PO_4_)_2_-PEG (Fig. 6a,b) exposed that the material acquires of mounded and aggregated with crumpled flakes intimately connected with each other and forming a higgledy-piggledy solid which look like transparent plates and silk flakes sheets. Somewhere its forming different and beautiful flowers like morphology of Cu_3_(PO_4_)_2_-PEG sample along with random flakes association. As can be observed from Fig. 6a the jasmine sort of structures to Fig. 6b the rose sort of structure. So the PEG block surfactant is giving a perfect nucleation foundation for the growth in solid state synthesis of the catalyst could be supposed.

Astoundingly, the RGO-Cu_3_(PO_4_)_2_-PEG catalyst fabricated by the identical course of action of solid state reaction is occupying a different and unique collections of flakes wrapped with a thin layers of materials was observed (Fig. 6c). As GO was added in the synthesis process and the reaction was left for the growth of the catalyst on it, that might helped the flakes of the catalyst Cu_3_(PO_4_)_2_-PEG to grow along with the thin layers of the graphene. And this is providing an extra flavor to the morphology in the matter of 8RGO-Cu_3_(PO_4_)_2_-PEG. The Fig. 6d is the magnified image of the later one which more precisely evident the well mannered growth of the catalyst along with the graphene oxide thin layers. This directly proofs the formation of the hybrid with RGO even in a solid state reaction procedure where GO was added directly to the reaction step without using any exfoliation or dispersion of GO before developing the semiconductor material on it as in liquid state reaction synthesis.

### Optical Behavior Analysis

**Figure 7:**
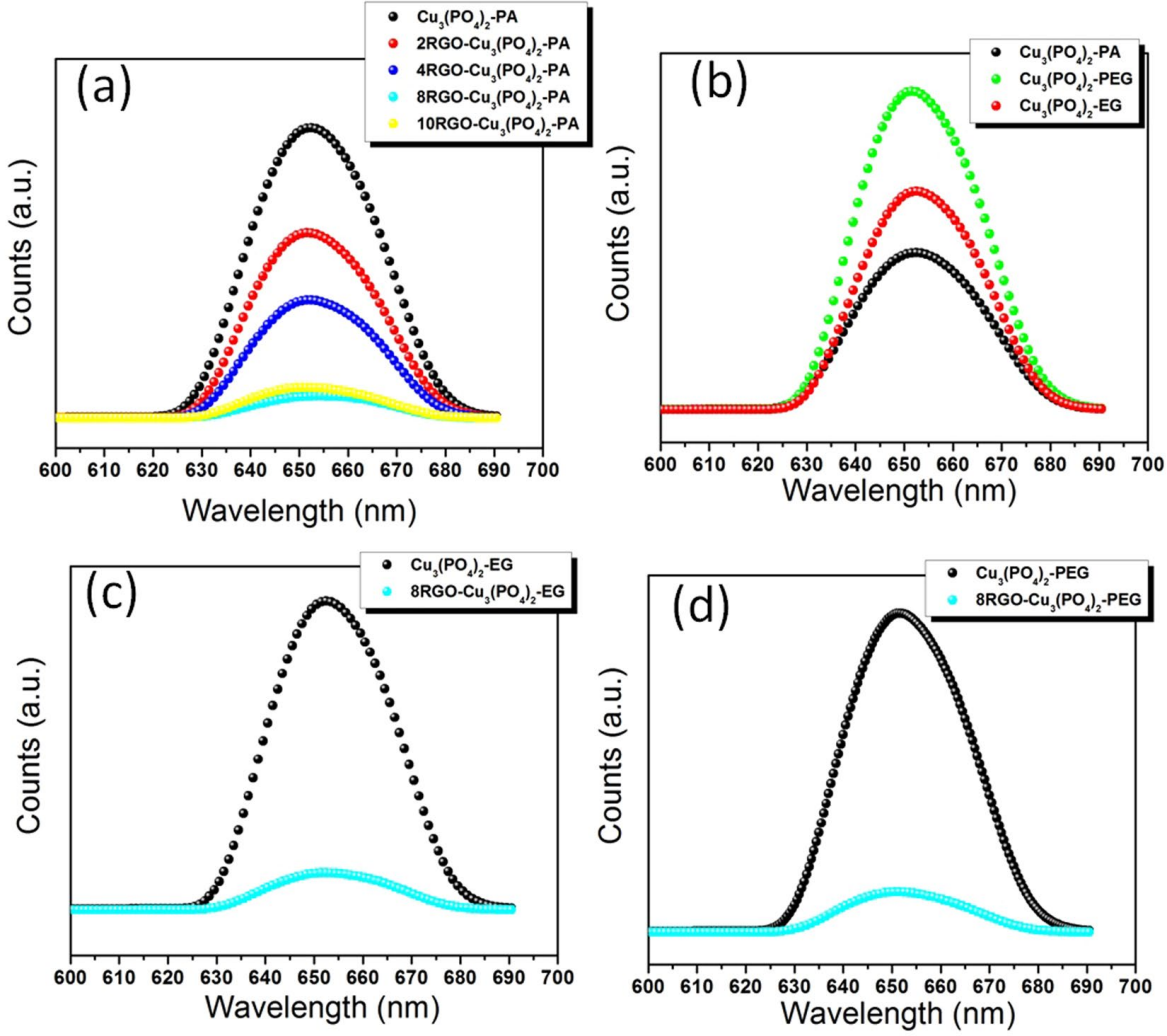
The PL spectra of (**a**) xRGO-Cu_3_(PO_4_)_2_-PA where x = 0, 2, 4, 8, 10 (**b**) pure Cu_3_(PO_4_)_2_-PA/PEG/EG catalysts (**c**) Cu_3_(PO_4_)_2_-EG and 8RGO-Cu_3_(PO_4_)_2_-EG (**d**) Cu_3_(PO_4_)_2_-PEG and 8RGO-Cu_3_(PO_4_)_2_-PEG.

Before starting the whole study of the work here, first of all synthesis and optimization of the semiconductor was done with the RGO concentrations. Where the 8 wt. % optimized RGO hybridized with Cu_3_(PO_4_)_2_-PA synthesized by the way of photoassisted method was found as the ultimate concentration to be made hybrid, which was described further in the activity experiment section further. In any photocatalytic experimentation the Photoluminescence (PL) spectra is the key ingredient to get knowledge about the best performing photocatalyst. As the PL spectra directly proves the fate of the electron-hole (e^−^-h^+^) pairs in a semiconductor after the irradiation of photon on it. The PL spectra of all the fabricated samples are illustrated in Fig. 7a–d, showing the emission bands due to the transition between the energy levels in the compound. The emission spectrum of all the Cu_3_(PO_4_)_2_ presents a broad band centered around 652 nm was achieved for all method (liquid/solid) based synthesized materials. Figure 7a illustrating the steady state PL spectra of xRGO-Cu_3_(PO_4_)_2_-PA synthesized and optimized with variable RGO concentrations with it. In the said RGO variables with Cu_3_(PO_4_)_2_-PA the zilch RGO composite have the highest intensity as expected. The intensity of PL changes with the RGO content, indicating a relationship between optoelectronic properties and RGO introduction. As could be observed from that the intensity is pulled down with the higher RGO quantity addition to the semiconductor. And as said earlier the 8RGO-Cu_3_(PO_4_)_2_-PA photocatalyst is showing the lowest intensity which directly evident the lowest recombination of the charge carriers. After the optimization with RGO concentration the copper phosphate catalyst was synthesized by the way of other liquid and solid state reactions as explained in details in the experimental paragraph. So the PL spectra of all three pure Cu_3_(PO_4_)_2_-PA/PEG/EG without RGO were done as shown in Fig. 7b. Among the pure form of the Cu_3_(PO_4_)_2_-PA/PEG/EG, the Cu_3_(PO_4_)_2_-PA is showing the lowest intensity as compared to Cu_3_(PO_4_)_2_-EG and then the Cu_3_(PO_4_)_2_-PEG is the highest. This could be explained as the rod shaped Cu_3_(PO_4_)_2_-PA making the aspect ratio of the charge carriers to a one direction and thus the charge carriers e^−^-h^+^ could avoid each other for a prolonged time which neglects the charge carriers recombination. Cu_3_(PO_4_)_2_-EG is showing the intermediate intensity of PL can be explained by the same concept but may be the e^−^-h^+^ are getting separated for less time in this case as compared to the rod shaped Cu_3_(PO_4_)_2_-PA. Lastly, the Cu_3_(PO_4_)_2_-PEG making a three dimensional structure, where the e^−^-h^+^ are confined in a three dimensional direction and hence the charge carrier are getting more opportunity to get recombined and hence it has the highest intensity of PL spectra as compared to other two materials.

After the optimization with 8 wt.% of RGO in the catalyst Cu_3_(PO_4_)_2_-PA, the other two catalysts were hybridized only with the same 8 wt.% as 8RGO-Cu_3_(PO_4_)_2_-PEG and 8RGO-Cu_3_(PO_4_)_2_-EG. The PL spectra of these were also done and as expected a large difference between the pure and 8 wt.% RGO modified hybrid was observed (Fig. 7c,d**)**. So from the PL spectra analysis we could look forward to that the Cu_3_(PO_4_)_2_-PA will show excellent activity as compared to the Cu_3_(PO_4_)_2_-EG and then Cu_3_(PO_4_)_2_-PEG composite with RGO.

**Figure 8:**
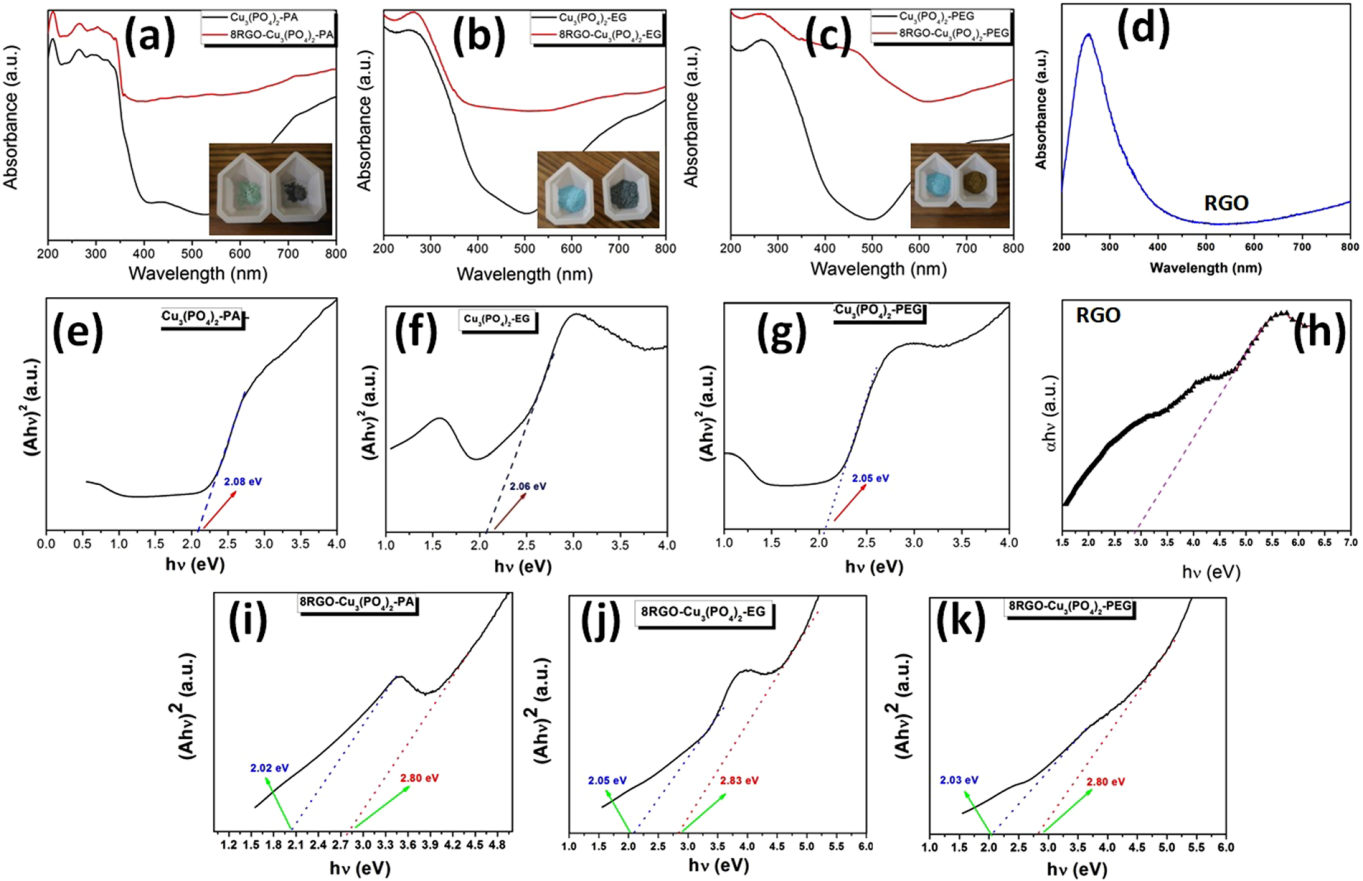
UV-vis diffuse reflectance spectra (DRS) of (**a**) Cu_3_(PO_4_)_2_-PA and 8RGO-Cu_3_(PO_4_)_2_-PA (**b**) Cu_3_(PO_4_)_2_-EG and 8RGO-Cu_3_(PO_4_)_2_-EG (**c**) Cu_3_(PO_4_)_2_-PEG and 8RGO-Cu_3_(PO_4_)_2_-PEG (**d**) RGO. (Inset: images showing the color of the respective samples). Band gap (Eg) determination for (**e**) Cu_3_(PO_4_)_2_-PA (**f**) Cu_3_(PO_4_)_2_-EG (**g**) Cu_3_(PO_4_)_2_-PEG (**h**) RGO (**i**) 8RGO-Cu_3_(PO_4_)_2_-PA (**j**) 8RGO-Cu_3_(PO_4_)_2_-EG and (**k**) 8RGO-Cu_3_(PO_4_)_2_-PEG samples using the Kubelka-Munk function of the diffuse reflectance (R) from Ultraviolet-visible diffuse reflectance spectra. The intercepts of the extrapolated straight lines give the corresponding band gaps of Cu_3_(PO_4_)_2_ and RGO in each hybrid.

Figure 8a–d shows the UV-vis diffuse reflectance absorption spectra of different samples. As shown in figures the pure copper phosphate samples whether it is solid/liquid based synthesized piece, it possessed photo-absorption from the UV to visible light until around 498 nm, corresponding to the band gap of 2.04–2.08 eV, which was calculated from the tangent line in the plot of the Kubelka-Munk function ((A*hν*)^2^) *vs*. photo energy (*hv*) by extrapolating the tangent lines in Fig. 8e–h^11,21,22^. Pure RGO also have sufficient absorption in the visible to Uv range as shown in figure. Again with the hybrid formation with 8 wt.% RGO, the intensity of the absorption was increased to many folds way far than the pure form of all. And which describes the hybrid formation with RGO traps more light (in a broader range) as compares to the pure samples in the light. This gives a clear suggestion about the visible light activity of the semiconductor hybrids. According to Fig. 8e–g, the band gaps from Cu_3_(PO_4_)_2_-PA, Cu_3_(PO_4_)_2_-EG and Cu_3_(PO_4_)_2_-PEG were estimated to be 2.08 eV, 2.04 eV and 2.05 eV, respectively. And RGO has bandgap with a value of 2.9 eV. Besides the pure copper phosphate band gap, we estimated that the hybrid structure of the RGO-Cu_3_(PO_4_)_2_ will show dual band gap in its Kubelka-Monk plot due to the interaction of two different semiconductors in it. So, as is expected, the whole copper phosphate/RGO series synthesized by liquid/solid based reaction with RGO are showing two band gaps at a time as marked in the Fig. 8i–k. 8RGO-Cu_3_(PO_4_)_2_-PA, is showing 2.02 eV and 2.80 eV band gaps of Cu_3_(PO_4_)_2_ and RGO, respectively. Similarly, the latter one the 8RGO-Cu_3_(PO_4_)_2_-EG is showing two band gaps 2.05 eV and 2.83 eV, respectively. Finally, 8RGO-Cu_3_(PO_4_)_2_-PEG are showing also two band gaps bearing values with 2.03 eV and 2.80 eV for Cu_3_(PO_4_)_2_ and RGO, respectively. Thus, it can be affirmed that there is an excellent interaction between the Cu_3_(PO_4_)_2_ and RGO whether its synthesized by solid or liquid state reaction.

**Figure 9:**
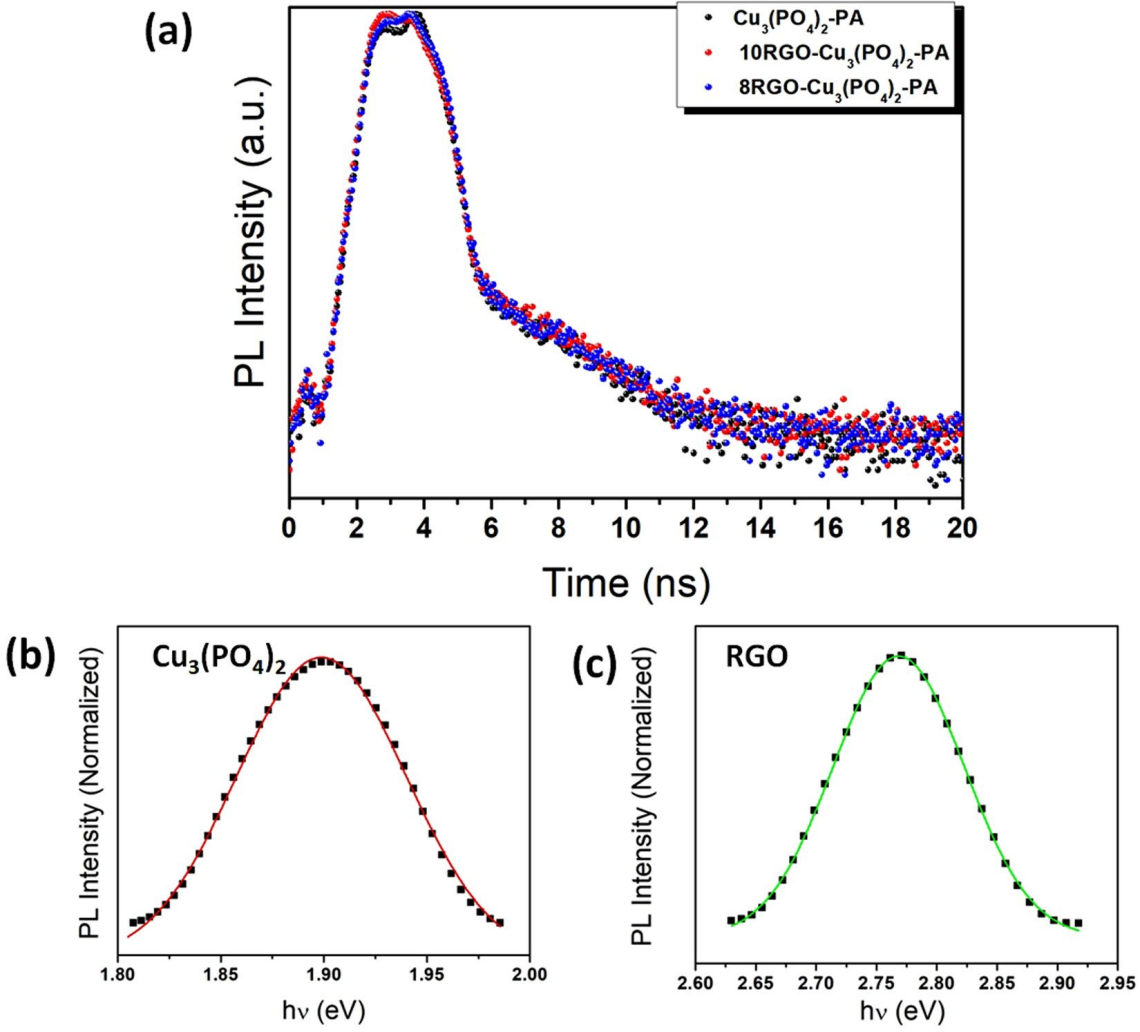
(**a**) Time-resolved photoluminescence decay spectra of Cu_3_(PO_4_)_2_-PA and 8 and 10 wt.% RGO-Cu_3_(PO_4_)_2_-PA heterojunction photocatalysts, at λ_ex_ = 470 nm. (**b**,**c**) PL spectrum of the Cu_3_(PO_4_)_2_-PA and RGO prepared hydrothermal reduction of GO. The symbols are the measured data and the solid line is the fit carried out.

Further the evidence of electron-hole separation in the heterojunction was characterized by time-resolved photoluminescence (Fig. 9a). Cu_3_(PO_4_)_2_, 8 and 10 wt.% RGO-Cu_3_(PO_4_)_2_-PA heterojunction photoluminescence decay were analyzed. In the photoluminescence decay Cu_3_(PO_4_)_2_-PA has the higher value of photoluminescence lifetime (τ = 0.233 ns) as compared to the heterojunction prepared with 8 and 10 wt.% RGO having photoluminescence lifetime, τ = 0.196 ns and τ = 0.198 ns, respectively. This result may be due to the higher rate of charge carrier recombination in case of bare Cu_3_(PO_4_)_2_-PA. The shorter lifetime of 8RGO-Cu_3_(PO_4_)_2_ as compared to bare Cu_3_(PO_4_)_2_-PA may be attributed to the better charge separation taking place at the heterointerface of RGO and Cu_3_(PO_4_)_2_ by providing nonradiative path for photogenerated charge carriers. Which further means that better charge separation occuring in the case of 8RGO-Cu_3_(PO_4_)_2_ leading to better photocatalytic activity of 8RGO-Cu_3_(PO_4_)_2_ than that of unmodified Cu_3_(PO_4_)_2_.

As observed above from Fig. 8e–k, the band gaps of pure copper phosphate and RGO were again reconfirmed by the normalized PL analysis as done by Tomm *et al*.^29,30^. Fig. 9b,c shows the PL intensity spectrum of the bare Cu_3_(PO_4_)_2_ and RGO maintaining a fit parameters. The solid line, which have matches the measured symbols very well (χ^2^ > 0.999 for RGO and 0.997 for Cu_3_(PO_4_)_2_). The energy parameter hν in figure can be interpreted as the optical band gap energy i.e. the lowest intrinsic exciton peak. Indeed, the value of 1.9 eV and 2.76 eV precisely coincides with the tauc plot data given in Fig. 8. However it’s obvious that the band gap calculated from photoluminescence intensity corresponding to emission maxima is lower than that of the original band gap. This is corroborating with our calculated band gap from tauc plot.

**Figure 10:**
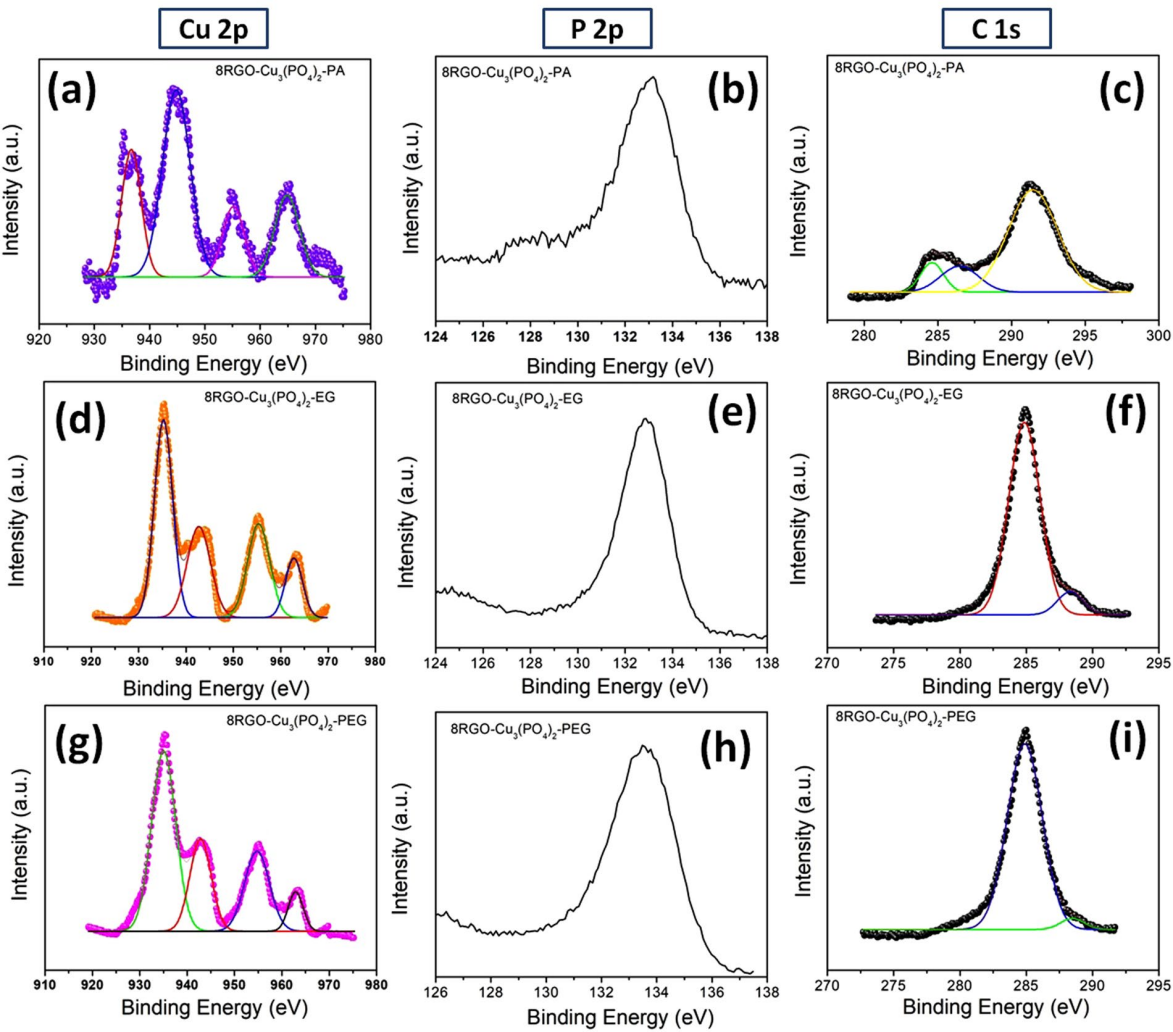
XPS survey spectra of 8RGO-Cu_3_(PO_4_)_2_-PA (**a**) Cu 2p (**b**) P 2p (gain the same elemental investigation) C 1 s, 8RGO-Cu_3_(PO_4_)_2_-EG (**d**) Cu 2p (**e**) P 2p (**f**) C 1 s and 8RGO-Cu_3_(PO_4_)_2_-PEG (**g**) Cu 2p (**h**) P 2p (**i**) C 1 s.

The XPS investigation was done to gain information on both the valence state of copper phosphate and the degree of reduction of graphene oxide as shown in Fig. 10. XPS spectra provided vast idea about the electronic structure and compositions of copper phosphate. The core peak of Cu 2p uncovers two main spin–orbit splitting at around 935.3 and 955.2 eV, corresponding to Cu 2p3/2 and Cu 2p1/2, which certifies the presence of Cu^2+^. The two peaks located at 942.6 and 962.7 eV are the “shake up” satellite of Cu 2p. Interestingly, all the morphology based copper phosphate are showing similar results which is certifying the +2 oxidation state of copper in the semiconductor (Fig. 10a,d,g). The P 2p scan peak located at around 133.1 eV evidences the presence of PO_4_^3−^ in the semiconductor (Fig. 10b,e,h).

XPS also used to evaluate the reduction level of RGO in the heterojunction, as the level of reduction is very crucial in quality of photocatalytic activity. The C 1 s peak (Fig. 10c,f,i) ranging from 280 to 292 eV in the XPS spectra, comprises peaks contributed by several oxygen functionalities that have different binding energies. The figure shows the C 1 s spectra of the RGO specimens present in the heterojunction. These spectra were deconvoluted and fitted using a symmetric Gaussian function. These peaks in Fig. 10c are corresponds to C-C at 284.6 eV, C-O at 286.5 eV and C=O at 291.2 eV for carboxylic acid or other oxygen functionalities. The proportion of the C-C group decreases while on the other hand, the proportion of the C=O group also decreases as compared to the pure GO^23^ indicating that the carboxyl functionalities got reduced by the irradiation of visible light during synthesis process of 8RGO-Cu_3_(PO_4_)_2_-PA. In case of 8RGO-Cu_3_(PO_4_)_2_-EG and 8RGO-Cu_3_(PO_4_)_2_-PEG also the reduction of GO to RGO could be evidenced from the C 1 s deconvluted peaks at 284.7 eV and 288.2 eV (Fig. 10f,i). This further confirms the reduction of graphene oxide during the synthesis process of heterojunction in liquid as well as solid state synthesis undoubtedly.

**Figure 11:**
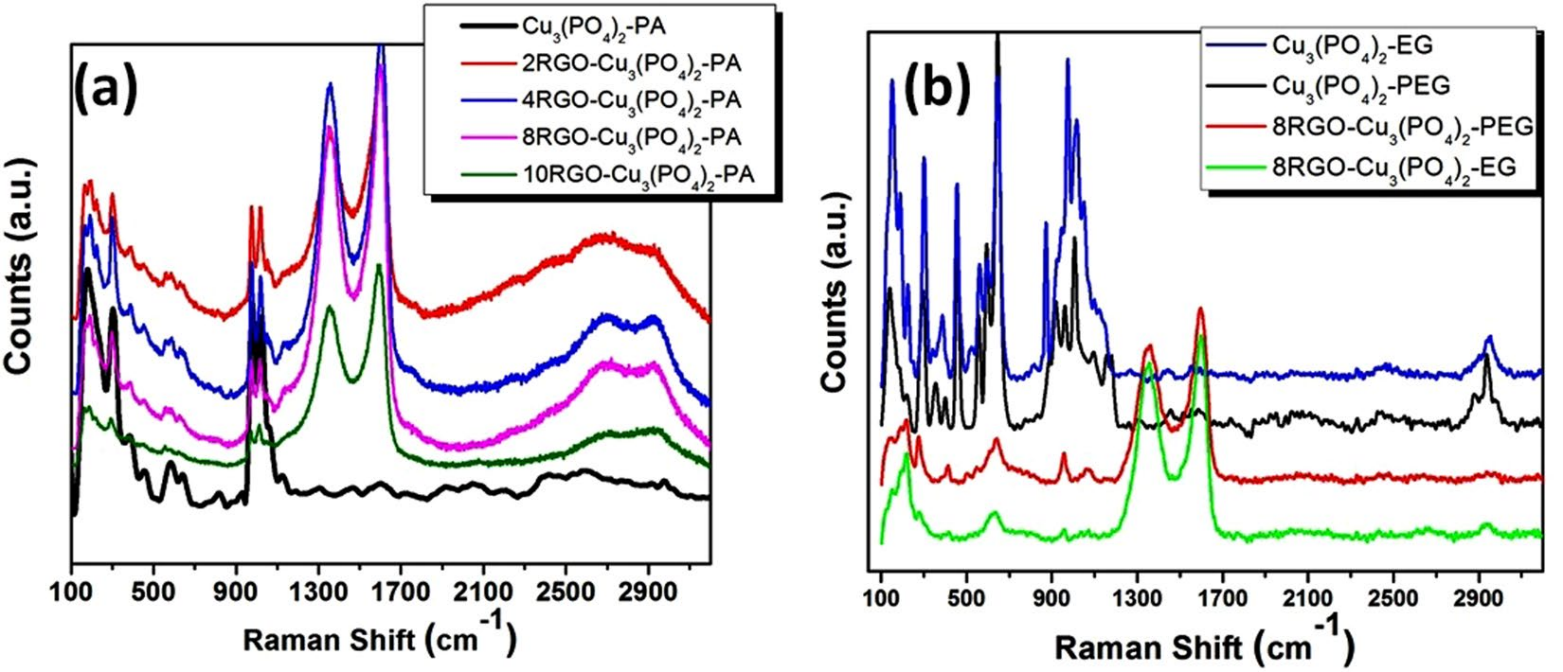
Raman spectra of (**a**) xRGO-Cu_3_(PO_4_)_2_-PA, (**b**) xRGO-Cu_3_(PO_4_)_2_-PEG/EG.

Raman spectroscopy is the most authenticate and reliable tool for the characterisation of graphene based materials, as it supply information and insights into the quality of graphene structure along with that it gives the idea about the presence of dopants in it. After the synthesis of the copper phosphate catalysts with 8 wt.% RGO, sharp bands appeared at 1351 cm^−1^, 1599 cm^−1^ which is assigned as D-band and G-band, respectively in 8RGO-Cu_3_(PO_4_)_2_-PA spectra (Fig. 11a). The prominent D-band at 1351 cm^−1^ is attributed to the sp^3^ defects in the sp^2^ lattice are related to the defects in graphene structure. Another peak at 2,709 cm^−1^ denoted by 2D-band is attributed to the development of graphene structure. While in 8RGO-Cu_3_(PO_4_)_2_-EG spectra, the D-band and G-band observed at 1353 and 1596 cm^−1^, respectively. And in the 8RGO-Cu_3_(PO_4_)_2_-PEG spectra, theses sharp peaks are noticed at 1359 and 1596 cm^−1^ (Fig. 11b). The Raman band intensity ratios (I_D_/I_G_) obtained in the matter of all RGO-copper phosphate samples are listed in Table 1. Where the solid state synthesized sample showing the ratio value as 0.80, and the EG and PA synthesized sample showing 0.83 and 0.84, correspondingly. Apart from this the Raman spectroscopy is also authenticates the lucrative combination of the RGO-copper phosphates whether by solid or liquid state reactions. In general, 2D bands of single-layer graphene sheets usually located at 2679 cm^−1^. But, the positions of the G and 2D bands shift into lower and higher wave numbers in case of multi-layer graphene sheets (including 2–6 layers) as reported by Akhavan *et al*.^31,32^. In our work, 2D band situated at 2697 cm^−1^ for bilayer sheet of graphene component in case of 8RGO-Cu_3_(PO_4_)_2_-PA with a 2D/G ratio value of ≈0.38 was observed. But in case of 8RGO-Cu_3_(PO_4_)_2_-EG sample a small 2D band located at 2674 cm^−1^ showing single layer of graphene in the heterojunction system.

**Table 1:** Intensity ratio of (I_D_/I_G_) in the copper phosphate samples.

Sample Name	I_D_/I_G_
8RGO-Cu_3_(PO_4_)_2_-PA	0.84
8RGO-Cu_3_(PO_4_)_2_-EG	0.83
8RGO-Cu_3_(PO_4_)_2_-PEG	0.80
8RGO-Cu_3_(PO_4_)_2_-PA (used catalyst, 4^th^ run)	0.83

### The Proton reduction potential of the catalyst

**Figure 12:**
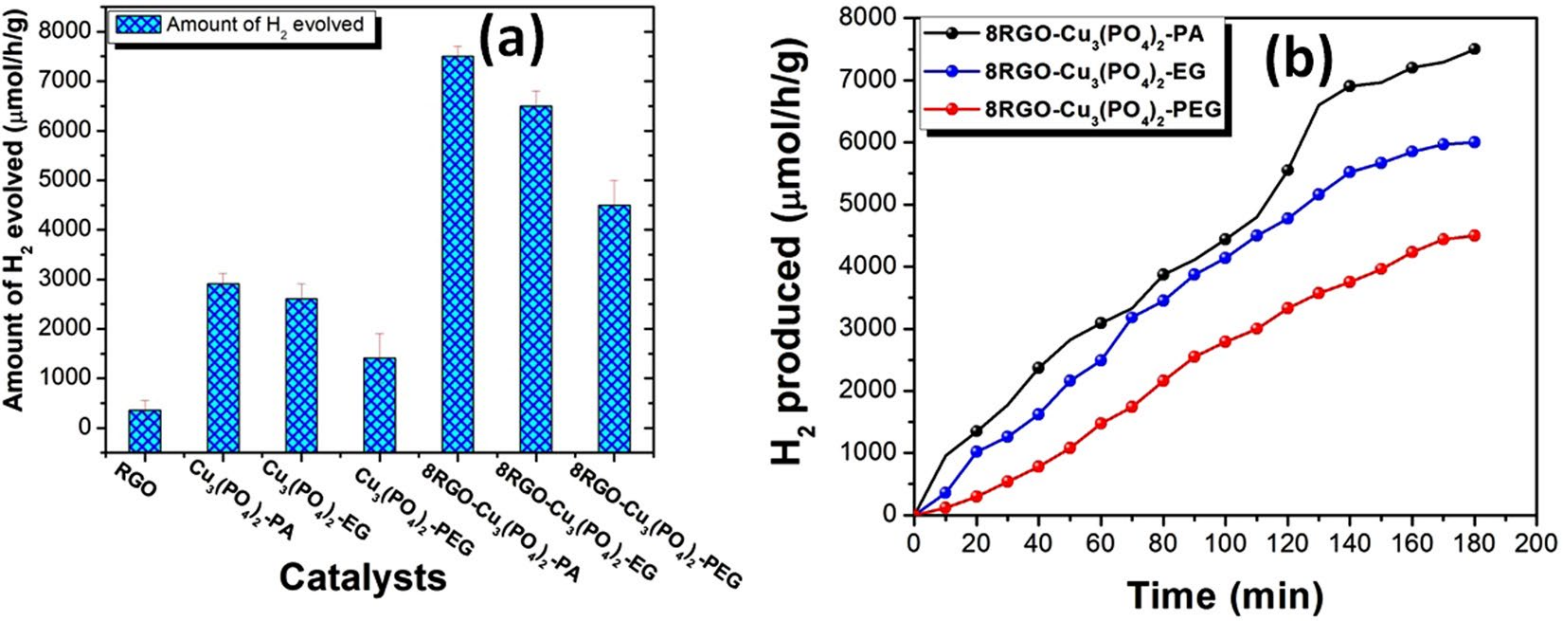
Proton reduction potential of (**a**) all the catalysts (**b**) the catalysts 8RGO-Cu_3_(PO_4_)_2_-PA/EG/PEG with respect to 180 min of time under visible light illumination.

The development of a noble metal free photocatalyst for proton reduction reaction is more vital as compared to inexpensive one. The noble metals including the Au, Ag, Pt and Pd incorporated with various metal oxides, metal oxide nanoarrays etc. to improve the photocatalytic reduction reaction of proton was established^33^. The steady-state H_2_ evolution rates are presented in Fig. 12. The use of RGO as another semiconductor electron donor in the heterojunction system resulted in an outstanding H_2_ production through reduction of proton in water. The bar graph Fig. 12a, showing the potential of all pure and RGO hybrid catalysts including the pure RGO as an individual catalyst. RGO is itself capable enough for the production of pure H_2_ by photosplitting of water as well described in our previous works^10,11,16,21–23^. Firstly, we have synthesized series of Cu_3_(PO_4_)_2_-PA with the RGO wt.% varying from 0, 2, 4, 8 and 10. From the photoactivity testing of this catalyst we searched out 8wt.%, as the most dynamic photocatalyst amongst all for generation of pure H_2_ from water under visible light photon. After optimization, we have synthesized the 8 wt.% of other two catalysts; i.e. 8RGO-Cu_3_(PO_4_)_2_-EG and 8RGO-Cu_3_(PO_4_)_2_-PEG. As can be seen from the bar graph that the photocatalysts independently showing aptitude for the production of H_2_ *Ca*. 2910, 2610 and 1410 µmol/h/g with Cu_3_(PO_4_)_2_-PA, Cu_3_(PO_4_)_2_-EG and Cu_3_(PO_4_)_2_-PEG, respectively under 180 min of visible light irradiation. For the hybrid junctions of 8RGO-Cu_3_(PO_4_)_2_-PA, 8RGO-Cu_3_(PO_4_)_2_-EG, 8RGO-Cu_3_(PO_4_)_2_-PEG, the amount of H_2_ generated were 7500, 6500 and 4500 µmol/h/g, respectively in the same reaction condition. Thus, this evidenced that RGO with the copper phosphate is actually working for the augmentation of the photoactivity and hence the charge carriers are living longer. Again, the Fig. 12b is showing the continuous increase in the rate of evolution of H_2_ with the optimized RGO hybrid catalysts. After photon irradiation all the catalysts are showing a homogeneous increase in the rate of the reaction with respect to the time.

**Figure 13:**
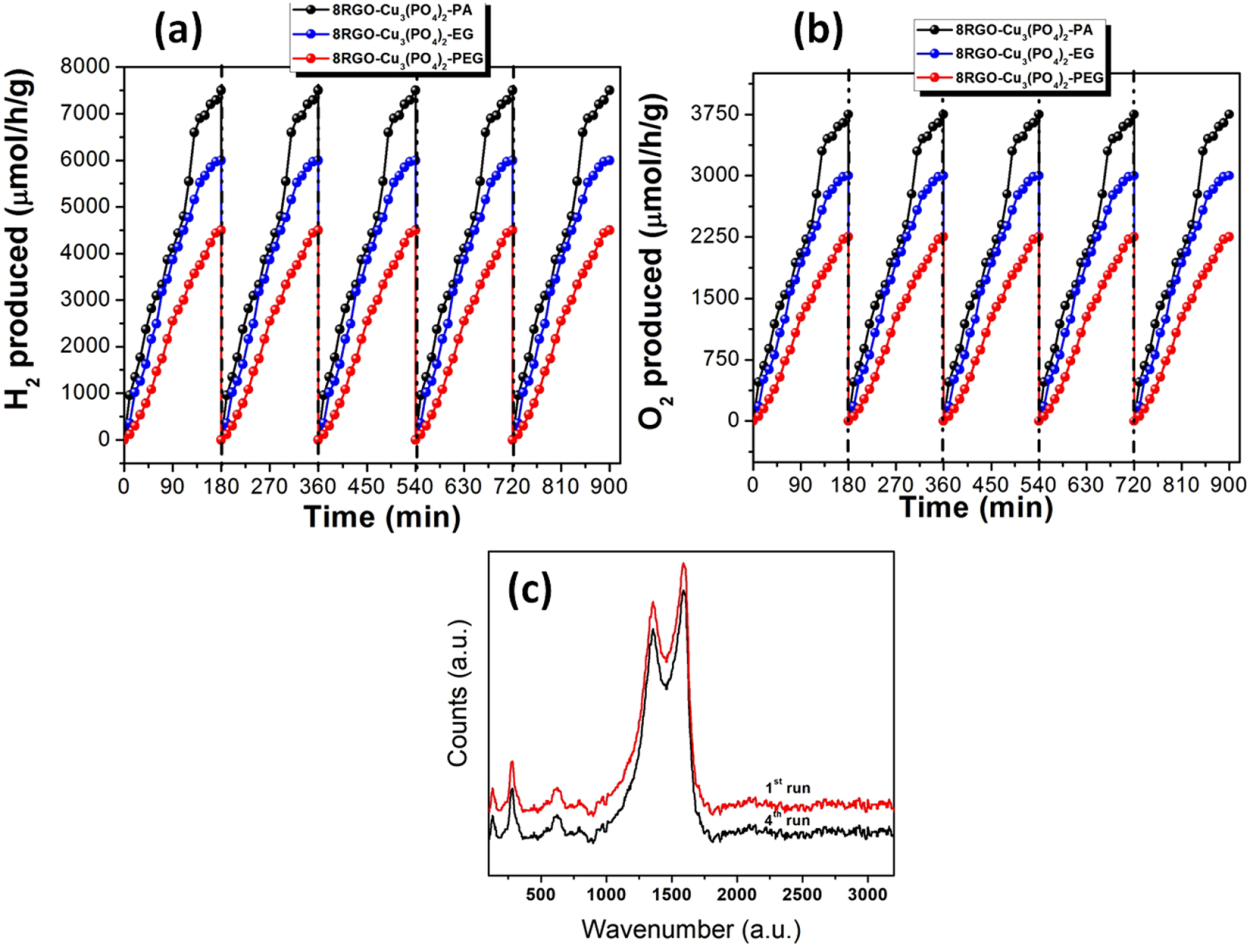
Reusability tests for the 8RGO-Cu_3_(PO_4_)_2_-PA/EG/PEG heterojunction photocatalysts to produce (**a**) H_2_ and (**b**) O_2_ via water splitting. Over every 180 mins the reaction system is bubbled with N_2_ for 15 min to remove the H_2_/O_2_ inside the reactor. (**c**) Comparison of raman spectra of pristine and used 8RGO-Cu_3_(PO_4_)_2_-PA after 4^th^ run (900 mins) during H_2_ production.

For any reaction, stability of the catalyst is the main concern for any future application of the desired catalyst. Thus, we have performed a stability as well as potential test of the catalysts for a prolonged period of time that is up to 900 mins both for the production of H_2_ and O_2_ by using sacrificial agents. Figure 13a,b is presenting the aptitude of the all three different RGO hybrid catalysts for the evolution of gases from water. As we can see there is not much difference in the amount of H_2_/O_2_ from the first run to the last 900 min timing reactions. In addition to that we have performed the raman analysis of the used catalyst 8RGO-Cu_3_(PO_4_)_2_-PA after 4^th^ run of H_2_ generation (Fig. 13c). As can be clearly seen the I_D_/I_G_ ratio is 0.83 (Table 1) which shows there is no such prominent increase of the carbonaceous defects^31,32^ after long irradiation time of 900 mins. This clearly confirms the non-photodegradation of RGO moiety in presence of Cu_3_(PO_4_)_2_-PA under light illumination. We could too able to synthesize roughly 2:1 ratio of the amount of H_2_:O_2_, which is feasible. Conclusively, we found that these catalysts are stable, non-photocorrosive (as we did not see any color in the reaction system after so many cycles), and are efficient candidates for both partial and overall water splitting reaction.

### The prediction of the mechanism

**Figure 14:**
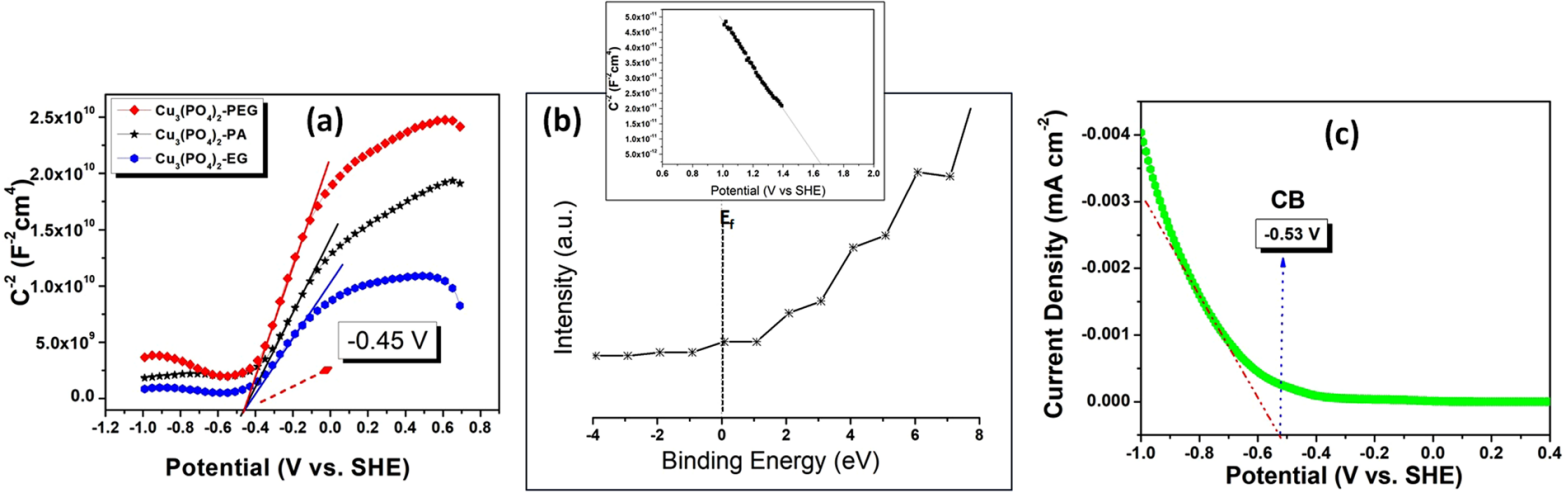
(**a**) Mott-Schottky plots for Cu_3_(PO_4_)_2_-PA, Cu_3_(PO_4_)_2_-EG, Cu_3_(PO_4_)_2_-PEG hybrid junction measured at 1 kHz *vs*. standard hydrogen electrode (SHE) scale. (**b**) XPS valence spectra of the pure reduced graphene oxide exhibiting a p-type semiconducting property (inset image: Mott-Schottky relationship for RGO with the applied potential in 0.5 M Na_2_SO_4_) (**c**) Cathodic linear potential scan converted to the SHE scale for determining the CB edge of the GO specimens at 5 mVs^−1^.

**Figure 15:**
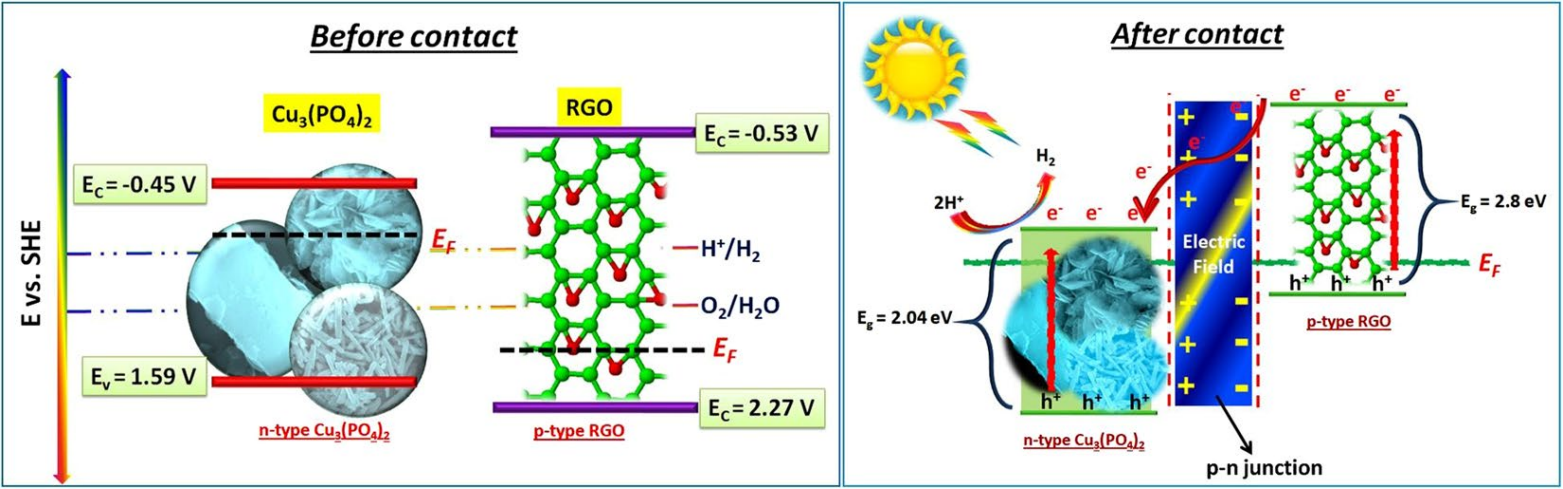
The band alignement of the catalysts before contact (left) and after contact (right) with p-n junction.

For a semiconductor photocatalyst to be qualified for the reduction of proton from water, two conditions are mandatory *Ca*. (1) the band gap of the semiconductor should be between 1.8 eV–3.0 eV to make the reaction feasible. (2) The conduction band (CB/E_c_) should more negative than the hydrogen reduction potential (0.0 V, NHE at pH 0). If the copper phosphate samples are capable enough to produce H_2_ then it should have E_c_ in a more negative value with respect to NHE/SHE. To know the position of the CB of the copper phosphate samples we have done the mott-schottky analysis of the catalysts. According to Allen J. Bard, the flat band potential of a semiconductor whether its n-type/p-type shows the approximate position of the conduction band for n-type and valence band for p-type semiconductors. Also Bard *et al*. described that the primary energetics of the system can be obtained from a knowledge of the flat-band potential (which for highly doped semiconductors approximately corresponds to the location of the conduction band edge, *Ec*, in n-type semiconductors and the valence band edge, *Ev*, in p-type**)**^34^. Wang *et al*.^35^ also determined the conduction band minimum (CBM) of *h*-BCN by Mott-Schottky method by the same way. So in order to find out the approximate position of the CB we have done the M-S analysis of the pure copper phosphate samples (Fig. 14a). From figure, the Mott-Schottky behavior is in accordance with the expected behavior of n-type semiconductor for all the three Cu_3_(PO_4_)_2_. When the negative slope is extrapolated to the x axis, a flat band value of −0.45 V versus SHE is obtained which we have considered as the conduction band minima position for the n-type Cu_3_(PO_4_)_2_ semiconductor. Again, it is notorious that RGO/GO is considered as p-type semiconductor as described in the introduction part. XPS valence spectra of RGO in Fig. 14b also shows that the RGO exhibits p-type semiconducting property which is in accordance with Okhavan *et al*.^36,37^. Again According to the Mott-Schottky equation, a linear relationship of 1/C^2^ vs E can be observed. The negative slope of the straight lines justifies the p-type conductivity of the RGO which is also in accordance with Yeh *et al*.^10^. In our previously reported work^22^, we have the GO specimens had E_C_ levels at −0.78 V versus Ag/AgCl, and in this work we have converted the value versus SHE to get a comparable energy levels for the description of the proper mechanism and we got the value of the CB of GO to be −0.53 V *vs*. SHE Fig. 14c. Notably, electrode potentials were converted to the SHE scale using E(SHE) = E(Ag/AgCl) + 0.199 V. Accordingly after determination of the CB of the individual photocataslysts, the valence band positions were calculated from the band gap achieved from the kubelka munk function (Fig. 9). Owing to the n-type conductivity of the copper phosphate and p-type conductivity of RGO, the mechanism of the photocatalytic activity of the hybrid junction was estimated in Fig. 15. Before connection p-RGO and n-Cu_3_(PO_4_)_2_ semiconductors normally have different positions of the Fermi levels. The positions of CB and VB edge potentials of n-Cu_3_(PO_4_)_2_ are −0.45 V and 1.59 V, respectively. For p-RGO, the CB and VB potentials are −0.53 V and 2.27 V, respectively (Fig. 15, left). However, the Fermi level of p-type RGO is moved up and the Fermi level of n-type Cu_3_(PO_4_)_2_ is moved down until an equilibrium state of Fermi levels (*E*_*f*_) is formed after contact (Fig. 15, right). Meanwhile, an inner electric field will be formed at the interface between p-type RGO and n-type Cu_3_(PO_4_)_2_. As shown in figure, the excited electrons on the CB of p-type RGO migrate easily to n-type Cu_3_(PO_4_)_2_ via the p-n junction interfaces; similarly, photo induced holes on the VB of n-type Cu_3_(PO_4_)_2_ surface transfer to p-type RGO owing to the different VB edge potentials. This transfer effectively suppresses the charge recombination, and thus results in superior photocatalytic performance. The photo-generated electrons can reduce H^+^ to form H_2_. Remaining hole then was scavenged by the methanol electron donating agent, meanwhile. As a result, the separation of the photogenerated carriers can be enhanced by the inner electric field. Thus, a larger amount of electrons accumulate on the surface of RGO and holes are left on the Cu_3_(PO_4_)_2_ surface, respectively as explained in the Fig. 15 (right). Also to examine the carrier density (N_D_) and flat band potential (V_fb_), we conducted capacitance measurements at 1 kHz frequency. The N_D_ and V_fb_ values for photoelectrodes in 0.1 M KOH electrolyte were determined using Mott-Schottky equations given below (eq. 1)^38^.

1 $$1/{{\rm{C}}}^{2}=2{/{\rm{\varepsilon }}{\rm{\varepsilon }}}_{0}{{\rm{A}}}^{2}e{{\rm{N}}}_{{\rm{D}}}({\rm{V}}-{{\rm{V}}}_{{\rm{fb}}}-{{\rm{k}}}_{{\rm{\beta }}}{\rm{T}}/e)$$

where C and A represent the interfacial capacitance and area, respectively, N_D_ is the number of donors, V is the applied voltage, k_β_ is the Boltzmann’s constant, T is the absolute temperature, and *e* is the electronic charge. The extrapolation of the linear part of the curves to (1/C^2^ = 0), gives the flat band potential of semiconductor. The N_D_ values were also determined from the slope of the linear part of the Mott-Schottky plot using following equation for Cu_3_(PO_4_)_2_-PA sample (eq. 2). Thus, the carrier density value of Cu_3_(PO_4_)_2_-PA was calculated as 2.611 × 10^19^ cm^−3^.

2 $${\rm{Slope}}=2/{\rm{\varepsilon }}{{\rm{\varepsilon }}}_{0}{{\rm{A}}}^{2}e{{\rm{N}}}_{{\rm{D}}}$$

## Conclusions

Solid state *vs*. liquid state synthesis of different morphological copper phosphate catalysts were synthesized successfully. The morphological structure was found to be rod, caramel treat like and also organized sheets/flower like. The rod shaped photocatalysts showed higher activity as compared to others as expected due to their high aspect ratio, large specific surface area, and excellent electronic or ionic charge transport than other two morphologies. By this a successful p-n hybrid junction was established by using p-type RGO for the first time. We have observed the amount of H_2_ generated 7500, 6500 and 4500 µmol/h/g over the p-n hybrid junction of 8RGO-Cu_3_(PO_4_)_2_-PA, 8RGO-Cu_3_(PO_4_)_2_-EG, 8RGO-Cu_3_(PO_4_)_2_-PEG, respectively under visible light illumination and the results are very much exhilarating as far as the cost and synthesis procedure is concerned.

## Methods

### Experimental Detail

All composites were characterized by XRD, diffuse reflectance (DR) UV/Vis, Raman, photoluminescence (PL) studies, TEM, energy-dispersive, X-ray spectroscopy (EDX), and electrochemical studies. XRD patterns were recorded on a Rigaku Miniflex (operated at 30 kV and 15 mA) powder diffractometer by using CuKα radiation with 2θ = 10–80° at a scanning rate of 5° min^−1^. The optical properties were examined using a UV/Vis spectrophotometer. DRUV/Vis spectra of the catalysts were recorded by using a Cary 100 spectrophotometer (Agilent) equipped with a DR accessory in the region of l = 200–800 nm, with boric acid as a reference. The Raman spectra were obtained by using a Renishaw Raman microscope (Model H33197), the excitation line of which was λ = 514 nm from an Arion laser. PL spectra were recorded with a LS 55 fluorescence spectrometer (Perkin–Elmer) with excitation at 380 nm at room temperature. The TEM images were obtained by means of an FEI TECNAI G2 model operated at 200 kV. Sampling for the study was done by dispersing the composites in 2-propanol using sonication for 3 min and then drop-drying on a copper grid coated with carbon film. Field emission (FE) SEM images were recorded on a Zeiss SupraTM 55 microscope. Prior to analysis, the samples were dispersed in isopropanol and drop dried over aluminum foil. To carry out PEC studies, thin films of Cu_3_(PO_4_)_2_ and 8RGO-Cu_3_(PO_4_)_2_ were made by using terpeneol and ethyl cellulose followed by calcinations of the films at 300 °C for 30 min and converted into an electrode by conventional methods. The PEC measurements were conducted in a three-electrode cell equipped with a quartz window and potentiostat-galvanostat (VersaStat3). The as-prepared films were used as the working electrodes. A 300 W xenon lamp (Newport, USA) equipped with a FSR-GG400 filter was used to irradiate the electrodes from the front side and was calibrated to 1 sun illumination (100 mWcm^−2^) by using a photodiode. Mott-Schottky (M-S; impedance) plots were obtained at a frequency of 1 kHz in the dark with an alternating current (AC) amplitude of 5 mV by using an IVIUM electrochemical workstation. The flat-band potential (Vfb) was determined and the carrier concentration were determined too. All electrochemical experiments were conducted under the above mentioned conditions, unless otherwise noted. All functional analyses were carried out in a cell with quartz windows. 0.1 M KOH for studies in (pH = 12.7) was taken as electrolyte for electrolytic experimentations. A three-electrode configuration was used, with a Pt electrode, counter electrode, a Ag/AgCl/saturated-KCl reference electrode (0.199 V NHE at 25 °C) and the synthesized catalysts as the working electrode. Time-resolved photoluminescence decay measurements were carried out using a time-correlated single-photon counting (TCSPC) spectrometer (Edinburgh, FLS 980). A diode laser (470 nm) was used as the excitation source. Time-resolved photoluminescence decay profiles were analysed using eq. 3. XPS measurements were performed on a XPS instrument (K-Alpha+, Make Thermo Fisher, UK) spectrometer with a non-monochromatized MgK_α_ X-ray source.

3 $$Fit={A}_{1}+{B}_{1}\cdot {e}(-t/{\tau }_{1})+{B}_{2}\cdot {e}(-t/{\tau }_{2})+{B}_{3}\cdot {e}(-t/{\tau }_{3})$$

### Photocatalytic experiments

#### Proton reduction reaction in water

In our group, we are working on the production of hydrogen in a purely photocatalytic system that uses common materials and can be operated continuously. Photocatalytic proton reduction reaction by water splitting was carried out in a Pyrex round-bottomed vessel by horizontal illumination of a 300 W xenon lamp source (Newport, USA) with λ > 400 nm cutoff filter (FSR-GG400). Water splitting was carried out by dispersing xRGO-Cu_3_(PO_4_)_2_ (0.05 g; x = 0–10 wt%) composites in an aqueous solution (50 mL) of methanol (10 vol%) as a sacrificial agent for H_2_ gas evolution and similar conditions as those maintained for O_2_ gas evolution, with a 0.03 m aqueous solution of AgNO_3_ (5 mL) as a sacrificial agent. Prior to visible-light illumination, the catalyst suspended in sacrificial agent was purged with N_2_. H_2_/O_2_ produced in the reaction was analyzed by GC (GC17A, Shimadzu) with a capillary molecular sieve (5 Å, Phenomenex) column in a thermal conductivity detector (TCD) with reference to standard H_2_ and O_2_ (purity: 99.995%).

### Synthesis

#### Materials

All reagents were of analytical grade and used without further purification. The natural graphite powder and all other reagents were purchased from Sigma-Aldrich. The fluorine-doped tin oxide (FTO) conductive glasses were cleaned ultrasonically with water, acetone and isopropanol successively in ultrasonic bath for 15 min.

#### Liquid State synthesis

For selectively liquid state synthesis of the copper phosphate catalyst was carried out by taking to respective solvents *Ca*. Water and Ethylene Glycol to get the different morphology. The detail procedure is described below. For the synthesis of RGO based copper phosphate samples the Graphene Oxide was taken as the precursor which was synthesized by modified hummers method as described in our previous work^22^.

**Figure 16:**
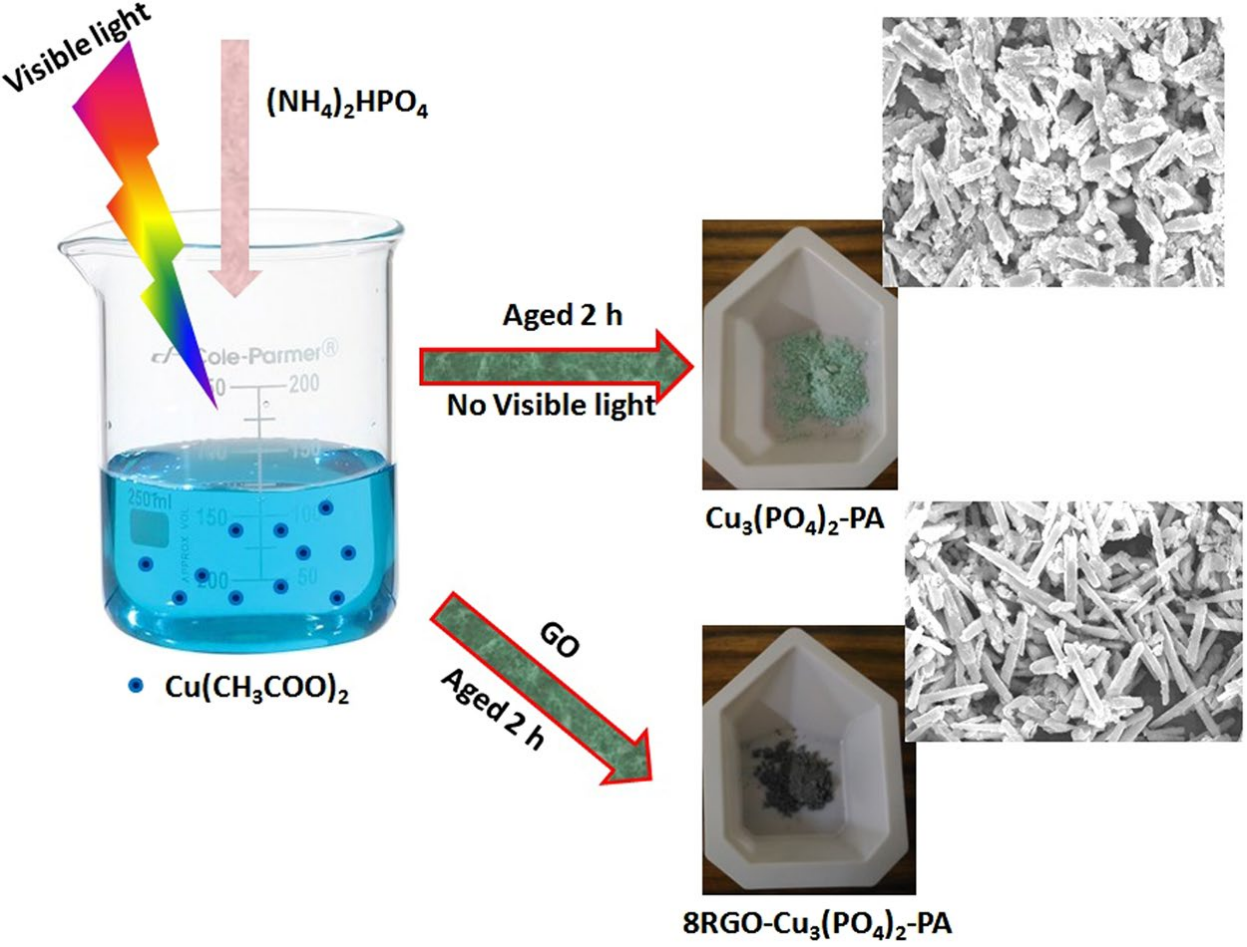
Pictorial representation of Cu_3_(PO_4_)_2_-PA synthesis.
- Synthesis with Ethylene Glycol as solvent:

- Photo-assisted synthesis with water as solvent:
  To obtain a rod like morphology of copper phosphate, 3 mmol of copper acetate was dissolved in water along with 2 mmol of triethanolamine to it to get a complete transparent solution. To this 2 mmol of (NH_4_)_2_HPO_4_ was added drop wise and the precipitate obtained was aged for 2 h. Then the slurry was washed and dried at 100 °C to get the Cu_3_(PO_4_)_2_-PA catalyst. Again xRGO-Cu_3_(PO_4_)_2_-PA catalysts (x = 0, 2, 4, 8, 10) were synthesized by adding different weight percentage of GO to the above procedure and aging the whole under the irradiation of visible light for 2 h with 5 mL of dry ethanol as the hole scavenger to get GO to RGO reduction during the synthesis procedure^21^. The schematic representation of the catalyst is shown in Fig. 16 along with the respective morphology.

**Figure 17:**
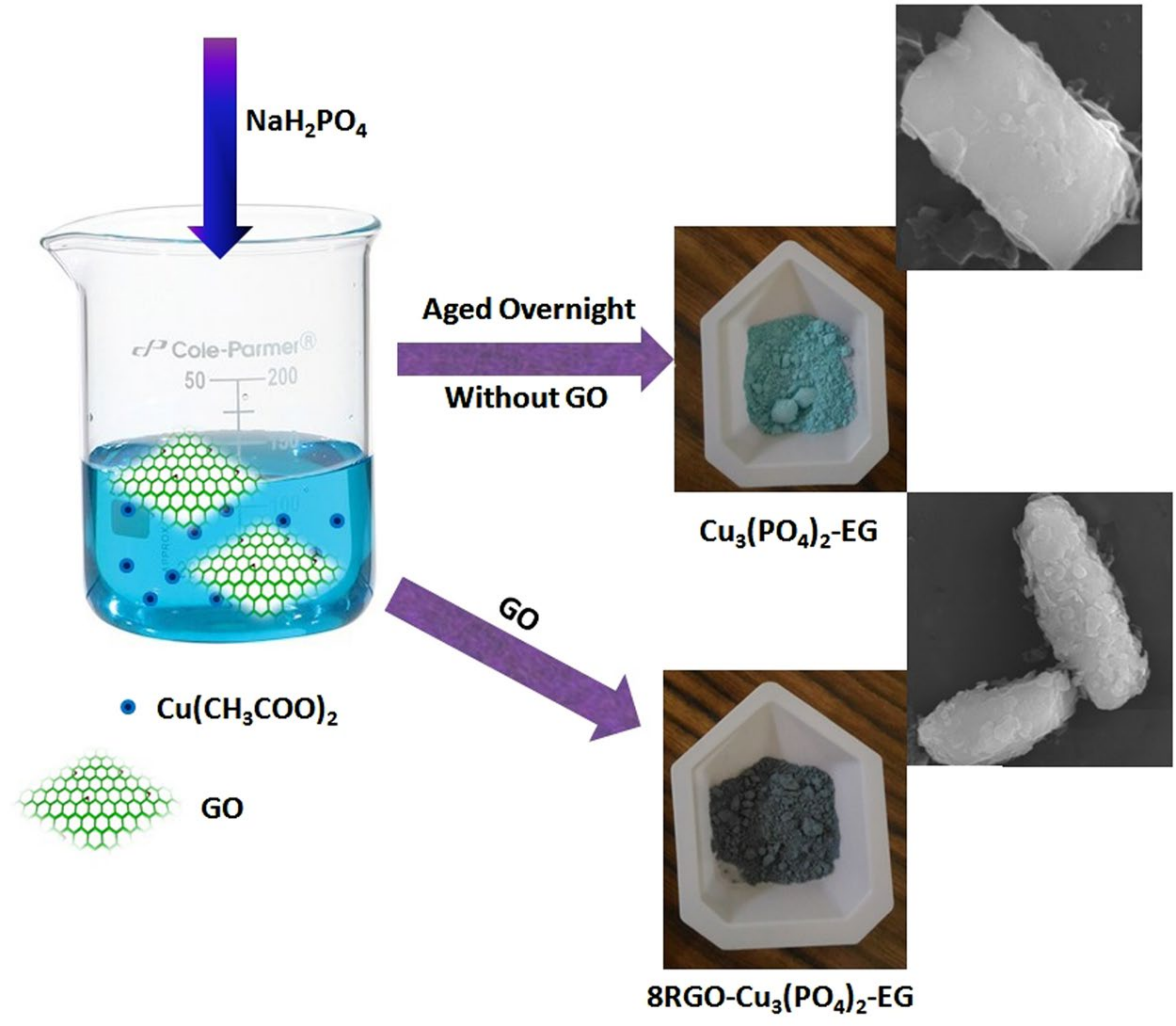
Pictorial representation of Cu_3_(PO_4_)_2_-EG synthesis.

The same copper phosphate catalyst was again synthesized by taking Ethylene glycol as solvent. First of all 1 M of copper acetate was taken in 10 mL of Ethylene Glycol and 2 mL of triethanolamine was added to it. To this solution 1 M of KH_2_PO_4_ was added drop wise. Then the whole mixture was aged overnight to get the complete precipitation. The precipitation was vigorously washed with ethanol and dried at 100 °C to get the Cu_3_(PO_4_)_2_-EG catalyst. To get 8RGO-Cu_3_(PO_4_)_2_-EG, stochiometric amount of GO first sonicated in 10 mL of Ethylene Glycol and then the whole procedure was repeated. The schematic representation of the catalyst is shown in Fig. 17 along with the respective morphology. The ethylene glycol solvent used here is a well known reducing agent which might reduces the GO to RGO during the synthesis process.

#### Solid state synthesis

**Figure 18:**
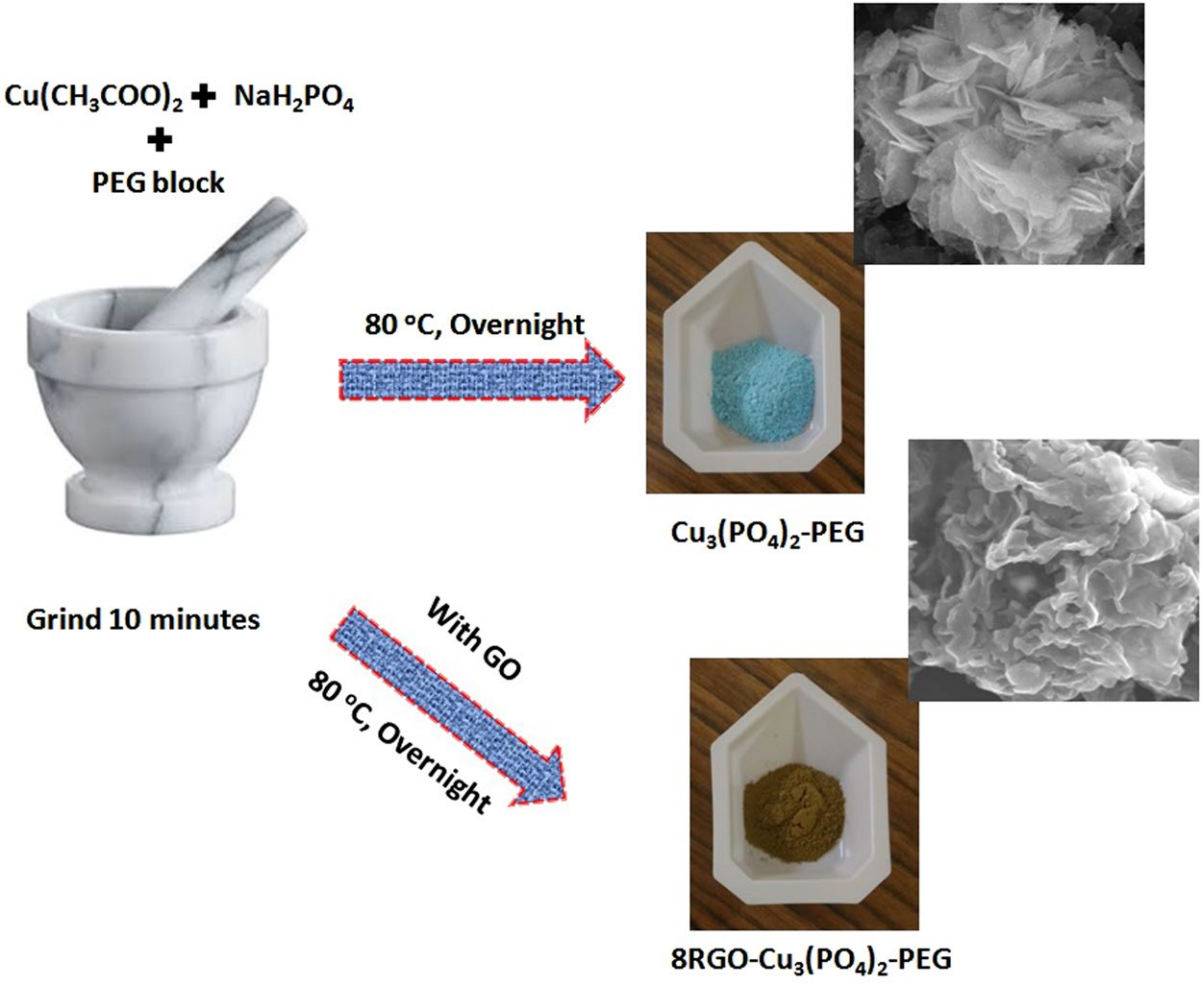
Pictorial representation of Cu_3_(PO_4_)_2_-PEG synthesis.

For selectively solid state synthesis of the copper phosphate catalyst, 1.816 g of copper acetate and 1.361 g of KH_2_PO_4_ were directly taken in a mortar pastel. To this stochiometric amount of Poly(ethylene glycol)-*block*-poly(propylene glycol)-*block*-poly(ethylene glycol) was added and the whole mixture was grinded until we got an uniform mixture. Then this mixture was kept in hot air oven at 60 °C overnight till the color of the mixture was changed from royal blue to a distinct blue color to obtain the phosphate catalyst. Then the obtained precipitates were washed vigorously with water followed by ethanol and dried at 100 °C. The 8RGO-Cu_3_(PO_4_)_2_-PEG was synthesized by following the similar procedure with addition of stochiometric amount of GO during the grinding step. The color change was noticed as from royal blue to brown color after the reaction. And again the PEG block is a strong reducing agent, so it can reduce the GO to RGO during the formation of hybrid junction. The schematic representation of the catalyst is shown in Fig. 18 along with the respective morphology.
